# Homologs and Transcriptional Isoforms of *Dorsal* Are Involved in the Response of *Apis cerana* to *Ascosphaera apis* Infection

**DOI:** 10.3390/biom16091223

**Published:** 2026-08-23

**Authors:** He Zang, Xinrui Chen, Xiang Li, Xue Yang, Qingwei Tan, Dafu Chen, Rui Guo, Jianfeng Qiu

**Affiliations:** 1 College of Animal Science, Fujian Agriculture and Forestry University, Fuzhou 350002, China; 2 College of Bee Science, Fujian Agriculture and Forestry University, Fuzhou 350002, China; 3 National & Local United Engineering Laboratory of Natural Biotoxin, Fuzhou 350002, China; 4 Apitherapy Research Institute of Fujian Agriculture and Forestry University, Fuzhou 350002, China

**Keywords:** *Apis cerana*, *dorsal*, Toll, antimicrobial peptides, alternative splicing

## Abstract

Chalkbrood disease caused by *Ascosphaera apis* threatens honey bee brood, yet the transcriptional mechanisms that coordinate antifungal immune responses in *Apis cerana* remain unclear. Here, RNA interference (RNAi) was used to examine two Dorsal homologs and selected *dorsal1* transcript isoforms during larval infection. *A. apis* inoculation increased *dorsal1*, *dorsal2*, and five antimicrobial peptide (AMP) transcripts in larval midguts, consistent with an infection-associated humoral response. Gene-level RNAi produced selective transcript responses: *dorsal1* knockdown was accompanied by reduced *apidaecin* and *defensin1* expression, whereas *dorsal2* knockdown was accompanied principally by reduced *defensin1* expression. *abaecin*, *defensin2*, and *hymenoptaecin* transcripts were not significantly altered after *dorsal* knockdown under the tested conditions. Isoform-targeted RNAi of RNA9886, RNA9888, and RNA9890 was likewise associated with distinct AMP transcript responses, with RNA9888 and RNA9890 more closely associated with *defensin1*. These transcript-level data support selective and partially overlapping Dorsal-associated regulation during the *A. cerana* larval response to *A. apis*, while direct differences in Dorsal protein abundance or activity remain to be established.

## 1. Introduction

Honey bees are essential pollinators of wild plants and agricultural crops, but their colonies are threatened by brood diseases that reduce larval survival and colony productivity [1,2]. Chalkbrood, caused by the filamentous fungal pathogen *Ascosphaera apis*, begins when larvae ingest spores that germinate in the gut and invade larval tissues [2]. *Apis mellifera* are highly susceptible to *A. apis*, whereas the Asian honey bee *Apis cerana* shows stronger resistance to this pathogen [3]. However, the molecular mechanisms that enable *A. cerana* larvae to mount an effective antifungal response remain incompletely defined. Recent transcriptomic and non-coding RNA studies have shown that *A. apis* infection reshapes immune and regulatory networks in *A. cerana* larvae [3,4], pointing to a need to connect these global responses with specific immune transcription factors.

Once fungal pathogens breach physical barriers such as the cuticle and peritrophic membrane, insects rely on cellular and humoral immune responses to restrict infection [2,5,6,7,8,9]. Antimicrobial peptides (AMPs), including *abaecin*, *apidaecin*, defensins, and *hymenoptaecin* in honey bees, are major effectors of the humoral response [10,11]. Recent honey bee work further indicates that gut-associated metabolites can prime humoral and cellular immunity, emphasizing the gut as an important immune-regulatory interface [12]. In insects, AMP transcription is primarily coordinated by NF-kappaB/Rel signaling pathways, especially Toll and immune deficiency (IMD) pathways [11,13,14,15,16]. The canonical Toll pathway is activated by fungal and Gram-positive bacterial cues and signals through Spatzle/Toll, MyD88, Tube, Pelle, and Cactus to release Dorsal/Dif family transcription factors, which then enter the nucleus and activate immune-effector genes [11,13,14,15,16,17]. Recent studies further support Toll signaling as a central determinant of antifungal defense while showing that Toll functions can vary across insect lineages and pathogen contexts [18,19,20]. In honey bees, the immune-gene repertoire is reduced relative to *Drosophila* but retains core Toll/IMD components [16]. Because the *Drosophila* Dif paralogue is absent or not clearly conserved in honey bees, Dorsal homologs are expected to be key NF-kappaB transcription factors linking Toll signaling to AMP expression [16,21]. In *A. mellifera*, RNAi-mediated silencing of *dorsal* genes reduces *defensin1* expression [21], but whether *A. cerana dorsal* homologs regulate AMP responses to *A. apis* remains unknown.

Alternative splicing (AS) provides an additional layer through which a single gene can generate transcript isoforms with different regulatory or functional potential. In insects, immune-related AS can expand receptor or effector diversity, as illustrated by pathogen-recognition genes such as *Dscam* in *Anopheles gambiae* [22,23]. In *A. cerana*, recent full-length transcriptomic work has shown that AS and alternative polyadenylation can respond to environmental stress and vary with worker age [24], indicating that isoform usage may be condition-dependent. In our previous full-length transcriptome of *A. cerana* larval guts [25], gene5492 and gene8512 were annotated as embryonic polarity protein dorsal (GenBank Accession No. 107996080; hereafter *dorsal1*) and embryonic polarity protein dorsal-like (GenBank Accession No. 107999258; hereafter *dorsal2*), respectively. The *dorsal1* gene produces seven transcript isoforms, whereas *dorsal2* produces a single transcript. These annotations raise an important unresolved question: whether *dorsal1* transcript isoforms are only structural variants or whether they contribute selectively to immune regulation during fungal infection.

RNA interference (RNAi) is a sequence-specific post-transcriptional silencing approach that has been widely used for functional studies in insects and honey bees [21,26,27,28,29,30,31]. Here, we used dsRNA feeding to silence *dorsal1*, *dorsal2*, and three selected *dorsal1* transcripts (RNA9886, RNA9888, and RNA9890) in *A. apis*-inoculated *A. cerana* larvae. Knockdown efficiency and the transcript abundance of five AMP genes—*abaecin*, *apidaecin*, *defensin1*, *defensin2*, and *hymenoptaecin*—were subsequently examined. We hypothesized that perturbing the two Dorsal homologs and individual *dorsal1* transcripts would be associated with distinct AMP transcript responses during fungal infection. This study therefore aimed to clarify the gene- and transcript-specific roles of Dorsal factors in the antifungal immunity of *A. cerana* larvae.

## 2. Materials and Methods

### 2.1. Insect Rearing and Fungal Inoculation

**Figure 1:**
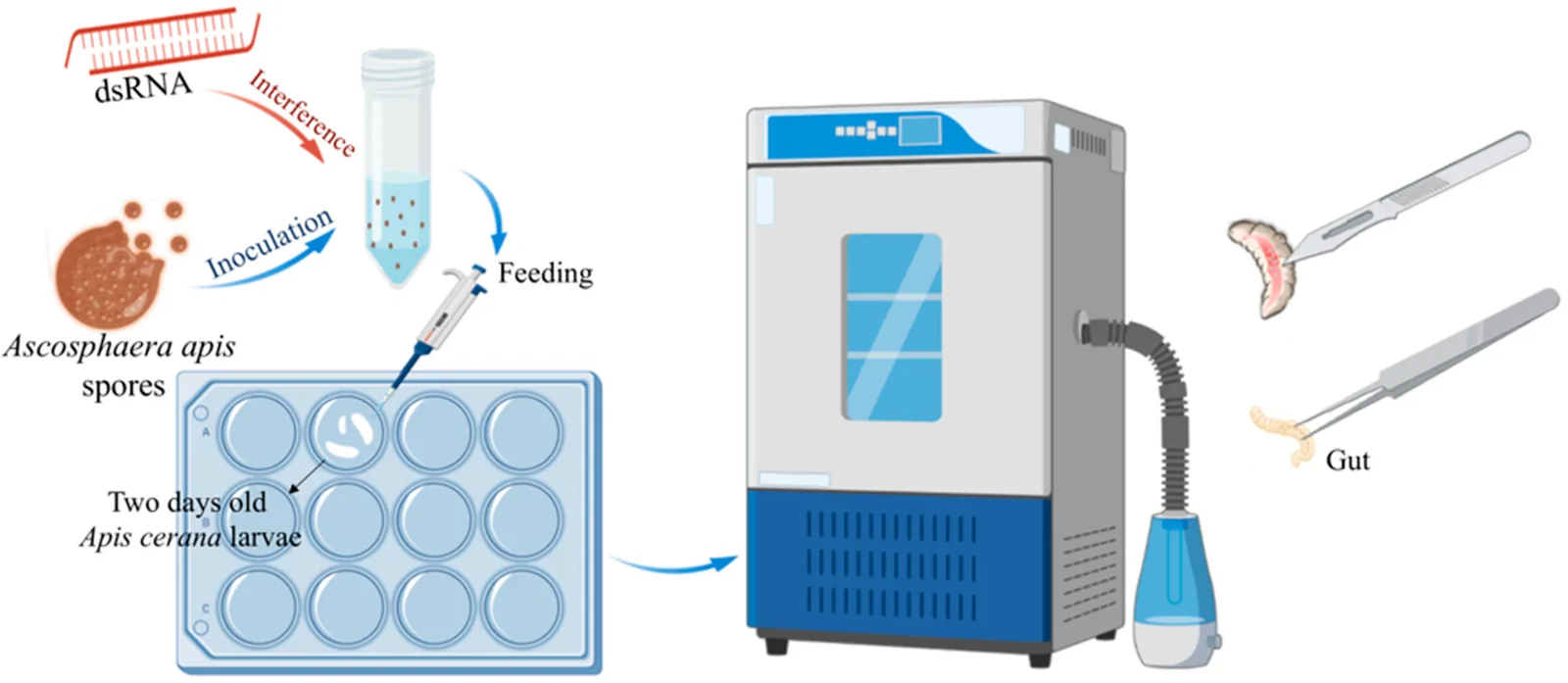
Schematic diagram illustrating the procedures for *Ascosphaera apis* spore and dsRNA feeding, as well as the collection of midgut samples from bee larvae.

*A. cerana* larvae used in this study were obtained from three colonies maintained in the teaching apiary of the College of Bee Science, Fujian Agriculture and Forestry University (Fuzhou, China). Each colony contributed one independent biological replicate to every experimental group. Before larval collection, all three colonies were inspected and showed no clinical signs of chalkbrood. Midguts from representative larvae were dissected by gently pulling out the alimentary tract, homogenized, and examined by light microscopy; no *A. apis* spores or other pathogen-like structures were observed. The colonies were therefore considered clinically healthy for larval collection. *A. apis* was originally isolated from chalkbrood mummies and deposited in the China General Microbiological Culture Collection Center (CGMCC No. 40895). Purified *A. apis* spores were prepared as described previously [32] and maintained in the Honey Bee Protection Laboratory. Larval diets were prepared according to Feng et al. [33], pre-warmed to 35 °C, and added to 48-well culture plates (Figure 1). Two-day-old larvae were transferred at one larva per well and maintained at 35 °C and 90% relative humidity. At 3 days of age, each larva in the infection group received a single 50 µL ration containing *A. apis* spores at 1 × 10^7^ spores/mL; control larvae received the same volume of spore-free diet. Larvae were observed individually, and only larvae that completely consumed the 50 µL treatment ration were retained. Fresh diet was supplied once daily thereafter, and dead larvae were removed every 24 h. Midguts were collected at the time points specified for each assay, immediately frozen in liquid nitrogen, and stored at −80 °C until RNA extraction. For each treatment and sampling time, *n* = 3 denoted three colony-derived biological replicates, each prepared by pooling the midguts of three larvae (nine larvae per treatment per time point).

### 2.2. Bioinformatic Analysis and Gene-Structure Visualization

The gene structures of *dorsal1* and *dorsal2* were visualized using Integrative Genomics Viewer (IGV) [34]. The *dorsal1* and *dorsal2* annotations were based on the full-length transcriptome and genome annotation described in our previous study [25], together with the corresponding GenBank annotations for *dorsal1* (accession No. 107996080) and *dorsal2* (accession No. 107999258). The transcript isoforms analyzed in this study included *dorsal1* isoforms RNA9886, RNA9888, RNA9889, RNA9890, RNA9891, ONT.3970.1, and ONT.3970.2, and the single annotated *dorsal2* transcript. The transcript sequences are provided in Supplementary Data S1.

### 2.3. dsRNA Design, Synthesis, and RNAi

Gene-specific dsRNA fragments were designed to target either shared or transcript-specific regions of *dorsal* genes. The dsRNA-dorsal1 fragment targeted a region shared by *dorsal1* transcript isoforms, whereas dsRNA-dorsal2 targeted *dorsal2*. Isoform-specific dsRNAs were designed for three *dorsal1* transcripts, RNA9886, RNA9888, and RNA9890. The approximate dsRNA fragment lengths were 254 bp for dsRNA-dorsal1, 232 bp for dsRNA-dorsal2, 252 bp for dsRNA-RNA9886, 203 bp for dsRNA-RNA9888, and 203 bp for dsRNA-RNA9890. Partial cDNA fragments were amplified using primers containing T7 promoter sequences (Table S1), ligated into the pClone007 vector (Tsingke, Shanghai, China), and transformed into *E. coli* DH5α. Recombinant plasmids were verified by Sanger sequencing and extracted using the FastPure Plasmid Mini Kit (Vazyme, Nanjing, China). In vitro transcription was performed using the T7 RNAi Transcription Kit (Vazyme, Nanjing, China) to generate dsRNA-dorsal1, dsRNA-dorsal2, dsRNA-RNA9886, dsRNA-RNA9888, dsRNA-RNA9890, and dsRNA-egfp, which served as the negative control.

RNAi was performed by feeding dsRNA to larvae. Two-day-old larvae were transferred to 48-well culture plates and maintained at 35 °C and 90% relative humidity. At 3 days of age, each larva received a single 50 µL treatment ration. In *A. apis*-inoculated RNAi assays, the spores and the corresponding dsRNA treatment were included in the same ration. Every dsRNA species was supplied at 20 ng/µL: single-dsRNA treatments therefore contained 20 ng/µL total dsRNA, paired treatments contained 20 ng/µL of each dsRNA (40 ng/µL total), and the triple transcript-targeting treatment contained 20 ng/µL of each dsRNA (60 ng/µL total). dsRNA-*egfp* at 20 ng/µL served as the negative control. The treatments included dsRNA-*egfp*, dsRNA-*dorsal1*, dsRNA-*dorsal2*, dsRNA-*dorsal1* plus dsRNA-*dorsal2*, dsRNA-RNA9886, dsRNA-RNA9888, dsRNA-RNA9890, and the indicated paired or triple transcript-targeting dsRNAs. Complete consumption of the treatment ration was confirmed for each retained larva, and fresh diet was supplied once daily thereafter. Midguts were collected at the time points indicated for each experiment, frozen in liquid nitrogen, and stored at −80 °C until analysis.

For the uninfected and *A. apis*-inoculated gene-level RNAi time courses, each treatment at each age (4, 5, and 6 days) comprised three colony-derived biological replicates of three pooled larval midguts (nine larvae per treatment per age; 27 larvae per treatment across the three-age time course). For the infection-only comparison at 6 days, each condition comprised three biological replicates of three pooled midguts (nine larvae per condition). For the gene-level AMP assay and the transcript-targeted RNAi assays, each treatment at 6 days likewise comprised three biological replicates of three pooled midguts (nine larvae per treatment).

### 2.4. RNA Extraction and cDNA Synthesis

Total RNA was extracted from larval midgut tissues using TRIzol reagent (Thermo Fisher Scientific, Waltham, Massachusetts, USA) according to the manufacturer’s instructions. RNA concentration and purity were assessed before reverse transcription. First-strand complementary DNA (cDNA) was synthesized using the Maxima H Minus Reverse Transcriptase Kit (Thermo Fisher Scientific, Waltham, Massachusetts, USA) following the manufacturer’s protocol. RNA and cDNA samples were stored at −80 °C until subsequent gene cloning and RT-qPCR analysis.

### 2.5. Real-Time Quantitative PCR (RT-qPCR)

RT-qPCR was performed to quantify the transcript abundance of *dorsal* genes, *dorsal1* transcript isoforms, and AMP genes under the indicated treatment conditions. Reactions were performed on a QuantStudio 3 Real-Time PCR System (Thermo Fisher Scientific, Waltham, Massachusetts, USA) using Hieff qPCR SYBR Green Master Mix (Yeasen, Shanghai, China). Each 20 µL reaction contained 10 µL of SYBR Green Master Mix, 1 µL of cDNA template, 1 µL of each primer, and 7 µL of double-distilled water. The amplification program was 95 °C for 5 min, followed by 34 cycles of 95 °C for 50 s, 55 °C for 30 s, and 72 °C for 50 s. Each colony-derived biological replicate was measured in three technical qPCR replicates. Amplification efficiencies were determined directly from the original cycle-by-cycle fluorescence data generated by the QuantStudio 3 system. The exponential phase of each amplification curve was evaluated by log-linear regression, and amplification efficiency was calculated from the resulting slope. The mean amplification efficiencies of all RT-qPCR assays ranged from 91.3% to 93.5%, with mean coefficients of determination (R^2^) ranging from 0.9984 to 0.9995. The reference gene actin-5C showed an amplification efficiency of 93.0 ± 4.0%, which was highly comparable to those of all target assays. Assay-specific amplification efficiencies and R^2^ values are provided in Table S1. The technical determinations were consolidated into one estimate for each biological replicate before statistical analysis and were not treated as independent observations. *actin-5C* (GenBank accession No. 107999330) was used as the endogenous reference because actin genes are commonly used as normalization controls in honey bee RT-qPCR studies [35,36]. For each sample, ΔCt was calculated as Ct_target_ − Ct_actin-5C_. For the *i*th colony-derived biological replicate, the treated sample was calibrated against the corresponding control sample from the same colony and experiment: ΔΔCt*_i_* = ΔCt_treatment,*i*_ − ΔCt_control,*i*_. The control was the uninoculated group or dsRNA-*egfp* group, as appropriate. Relative transcript abundance was calculated as 2^−ΔΔCt^ [37], which is equivalent to the ratio 2^−ΔCt(treatment)^/2^−ΔCt(control)^. Thus, calibrator scaling was applied only after within-sample target/reference normalization, and a single group-mean control was not imposed across the three colonies. Primer sequences and their applications are listed in Table S1. The available assay information is reported with reference to the MIQE 2.0 framework [38].

### 2.6. Data Analysis

Data are presented as the mean ± SD from three independent colony-derived biological replicates (*n* = 3); each biological replicate comprised three pooled larval midguts. The effects of larval age and dsRNA treatment on *dorsal1* or *dorsal2* expression were analyzed using two-way analysis of variance (ANOVA), followed by Sidak multiple-comparisons tests. The single prespecified contrast between *A. apis*-inoculated and uninoculated larvae was analyzed separately for each gene using Student’s *t*-test. Only prespecified comparisons between each dsRNA treatment and the dsRNA-*egfp* control were evaluated using Student’s *t*-test; no inferential comparisons among the active dsRNA treatments were made. Statistical analyses and figure preparation were performed using GraphPad Prism software (version 11). Differences were considered statistically significant at *p* < 0.05. Given the three-replicate design and the presence of multiple planned treatment-control contrasts, the effect magnitudes were not used to rank mechanistic potency among transcript targets.

## 3. Results

### 3.1. Analysis of the Dorsal Genes and Isoforms and dsRNA-Mediated Silencing of dorsal1 and dorsal2

**Figure 2:**
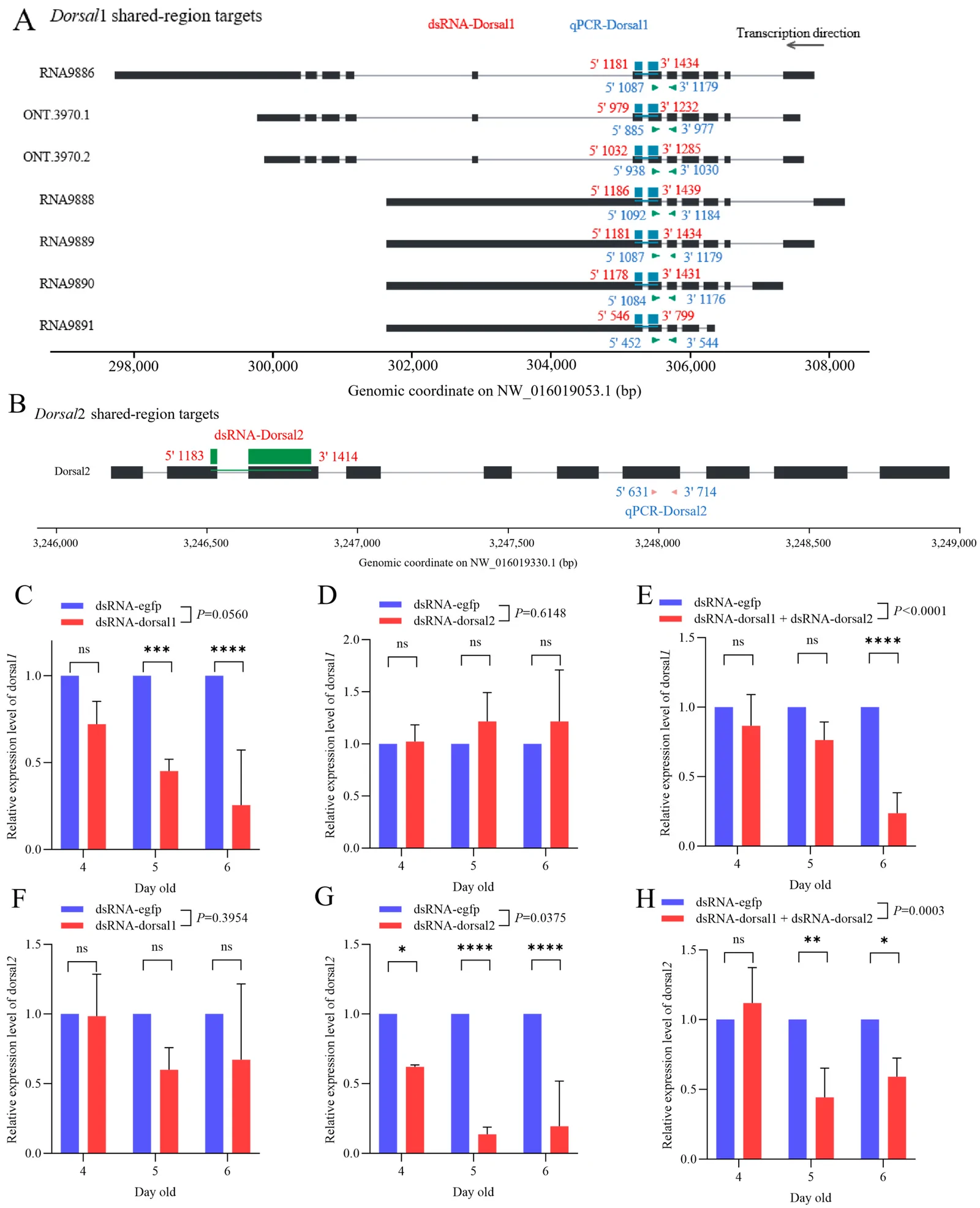
Design of shared-region dsRNA targets and validation of *dorsal1* and *dorsal2* knockdown in uninfected *A. cerana* larvae. (**A**) Locations of the dsRNA-dorsal1 target region and qPCR primer regions across *dorsal1* transcript isoforms. (**B**) Locations of the dsRNA-dorsal2 target region and qPCR primer region in *dorsal2*. (**C**–**H**) Relative expression of *dorsal1* and *dorsal2* in 4-, 5-, and 6-day-old larvae after dsRNA feeding: *dorsal1* after dsRNA-dorsal1 (**C**), dsRNA-dorsal2 (**D**), or dsRNA-dorsal1 plus dsRNA-dorsal2 (**E**), and dorsal2 after dsRNA-dorsal1 (**F**), dsRNA-dorsal2 (**G**), or dsRNA-dorsal1 plus dsRNA-dorsal2 (**H**). dsRNA-egfp was used as the control. Data are shown as mean ± SD (*n* = 3). Statistical significance was analyzed by two-way ANOVA followed by Sidak multiple comparisons. ns, *p* > 0.05; *, *p* < 0.05; **, *p* < 0.01, ***, *p* < 0.001, ****, *p* < 0.0001.

Gene-structure analysis showed that *dorsal1* generated seven transcript isoforms through alternative splicing, including RNA9886, RNA9888, RNA9889, RNA9890, RNA9891, ONT.3970.1, and ONT.3970.2 (Figure 2A). RNA9886, ONT.3970.1, and ONT.3970.2 each contained 11 exons, whereas the other dorsal1 transcripts differed mainly in the 5′ untranslated region, 3′ untranslated region, and the eighth exon. In contrast, *dorsal2* produced a single transcript containing 10 exons (Figure 2B).

To validate the RNAi strategy, dsRNA and RT-qPCR target regions were mapped onto *dorsal1* and *dorsal2*. The dsRNA-dorsal1 fragment targeted a region shared by the *dorsal1* transcript isoforms, whereas dsRNA-dorsal2 targeted the single *dorsal2* transcript (Figure 2A,B). In uninfected larvae, dsRNA-dorsal1 reduced *dorsal1* expression to 0.721, 0.451, and 0.255 of the dsRNA-egfp control at 4, 5, and 6 days old, corresponding to knockdown efficiencies of 27.9%, 54.9%, and 74.5%, respectively (Figure 2C). The reductions were significant at 5 and 6 days old (*p* = 0.0012 and *p* = 0.0001, respectively), whereas the 4-day-old reduction was not significant (*p* = 0.1133). dsRNA-dorsal2 did not reduce *dorsal1* expression at any time point (*p* = 0.9993, *p* = 0.6467, and *p* = 0.6464 at 4, 5, and 6 days old, respectively; Figure 2D), indicating target specificity. Co-administration of dsRNA-dorsal1 and dsRNA-dorsal2 reduced dorsal1 expression to 0.866, 0.763, and 0.236 of the control at 4, 5, and 6 days old, corresponding to 13.4%, 23.7%, and 76.4% knockdown, respectively; this reduction was significant at 6 days old (*p* < 0.0001) but not at 4 or 5 days old (*p* = 0.4958 and *p* = 0.1008; Figure 2E). Conversely, dsRNA-dorsal1 did not significantly reduce *dorsal2* expression (*p* = 0.9998, *p* = 0.2348, and *p* = 0.3887; Figure 2F), whereas dsRNA-dorsal2 reduced *dorsal2* expression to 0.621, 0.137, and 0.194 of the control at 4, 5, and 6 days old, corresponding to 37.9%, 86.3%, and 80.6% knockdown, respectively (*p* = 0.0140, *p* < 0.0001, and *p* < 0.0001; Figure 2G). The combined dsRNA treatment reduced *dorsal2* expression at 5 and 6 days old, with expression levels of 0.443 and 0.590 of the control, corresponding to 55.7% and 41.0% knockdown (*p* = 0.0016 and *p* = 0.0143, respectively), while no reduction was detected at 4 days old (*p* = 0.7120; Figure 2H).

**Figure 3:**
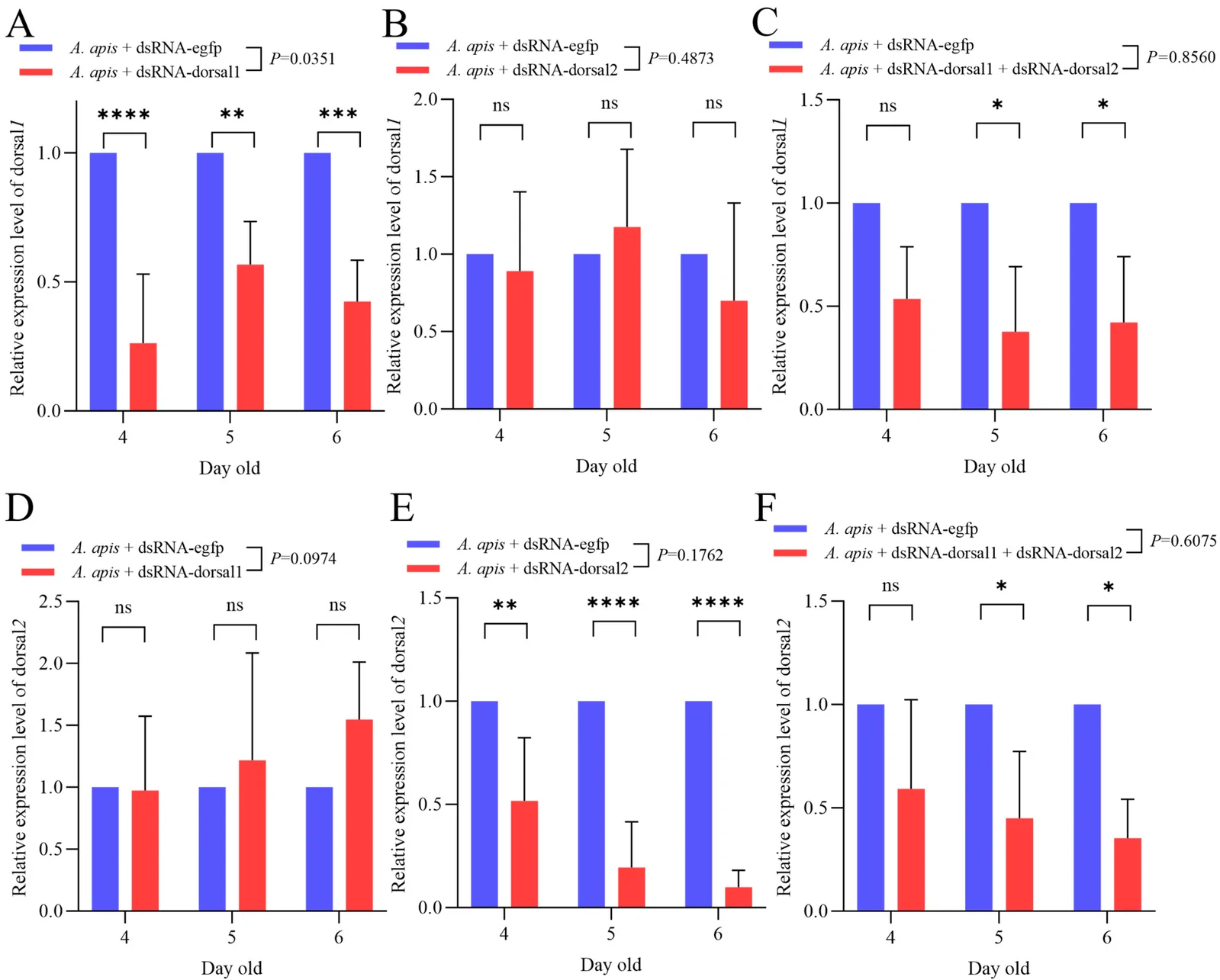
Validation of *dorsal1* and *dorsal2* knockdown in *A. apis*-inoculated *A. cerana* larvae. Relative expression of *dorsal1* and *dorsal2* was measured in 4-, 5-, and 6-day-old larvae after *A. apis* inoculation and dsRNA feeding. Panels show *dorsal1* expression after dsRNA-dorsal1 (**A**), dsRNA-dorsal2 (**B**), or dsRNA-dorsal1 plus dsRNA-dorsal2 (**C**), and dorsal2 expression after dsRNA-dorsal1 (**D**), dsRNA-dorsal2 (**E**), or dsRNA-dorsal1 plus dsRNA-dorsal2 (**F**). dsRNA-egfp was used as the control, and *Actin-5C* was used as the endogenous reference gene. Data are shown as mean ± SD (*n* = 3). Statistical significance was analyzed by two-way ANOVA followed by Sidak multiple comparisons. ns, *p* > 0.05; *, *p* < 0.05; **, *p* < 0.01, ***, *p* < 0.001, ****, *p* < 0.0001.

After *A. apis* inoculation, the target-specific knockdown patterns were retained (Figure 3). dsRNA-dorsal1 reduced *dorsal1* expression to 0.262, 0.568, and 0.424 of the dsRNA-egfp control at 4, 5, and 6 days old, corresponding to 73.8%, 43.2%, and 57.6% knockdown, respectively; all three reductions were significant (*p* = 0.0001, *p* = 0.0097, and *p* = 0.0011; Figure 3A). dsRNA-dorsal2 did not significantly reduce *dorsal1* expression (*p* = 0.9818, *p* = 0.9308, and *p* = 0.7425; Figure 3B). The combined dsRNA-dorsal1 and dsRNA-dorsal2 treatment reduced *dorsal1* expression to 0.536, 0.377, and 0.422 of the control at 4, 5, and 6 days old, corresponding to 46.4%, 62.3%, and 57.8% knockdown, respectively; the reductions were significant at 5 and 6 days old (*p* = 0.0102 and *p* = 0.0165) and marginal at 4 days old (*p* = 0.0558; Figure 3C). In parallel, dsRNA-dorsal1 did not reduce *dorsal2* expression in infected larvae (*p* = 0.9999, *p* = 0.9266, and *p* = 0.4493; Figure 3D), whereas dsRNA-dorsal2 reduced *dorsal2* expression to 0.517, 0.195, and 0.100 of the control, corresponding to 48.3%, 80.5%, and 90.0% knockdown, respectively (*p* = 0.0083, *p* = 0.0001, and *p* < 0.0001; Figure 3E). The combined dsRNA treatment reduced *dorsal2* expression to 0.592, 0.450, and 0.353 of the control, corresponding to 40.8%, 55.0%, and 64.7% knockdown, respectively; this effect was significant at 5 and 6 days old (*p* = 0.0397 and *p* = 0.0156) but not at 4 days old (*p* = 0.1509; Figure 3F).

### 3.2. Effects of dorsal1 and dorsal2 Knockdown on AMP Gene Expression After A. apis Infection

**Figure 4:**
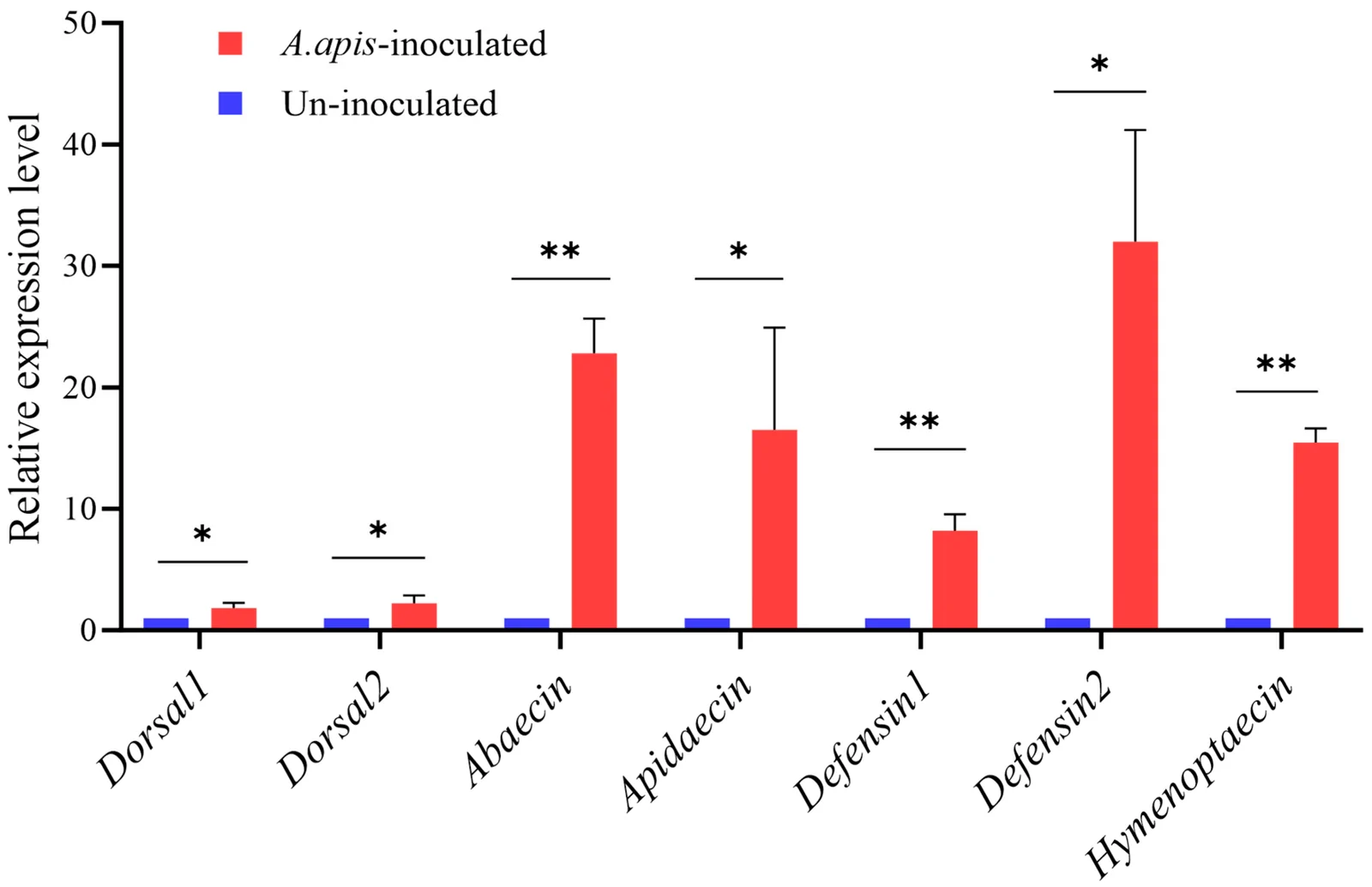
Expression of *dorsal* and AMP genes in *A. apis*-inoculated larvae. Relative transcript levels of *dorsal1*, *dorsal2*, *abaecin*, *apidaecin*, *defensin1*, *defensin2*, and *hymenoptaecin* were measured in the midguts of 6-day-old *A. apis*-inoculated larvae and uninoculated controls. Data are shown as mean ± SD (*n* = 3). Statistical significance was analyzed by Student’s *t*-test for each gene; *, *p* < 0.05; **, *p* < 0.01.

RT-qPCR analysis of 6-day-old larval midguts showed that *A. apis* inoculation was associated with increased *dorsal* and AMP transcript abundance. Compared with uninoculated larvae, infected larvae showed increased expression of *dorsal1* (*p* = 0.0417), *dorsal2* (*p* = 0.0397), *abaecin* (*p* = 0.0029), *apidaecin* (*p* = 0.0432), *defensin1* (*p* = 0.0057), *defensin2* (*p* = 0.0141), and *hymenoptaecin* (*p* = 0.0011) (Figure 4). These results document an infection-associated increase in the measured immune transcripts.

**Figure 5:**
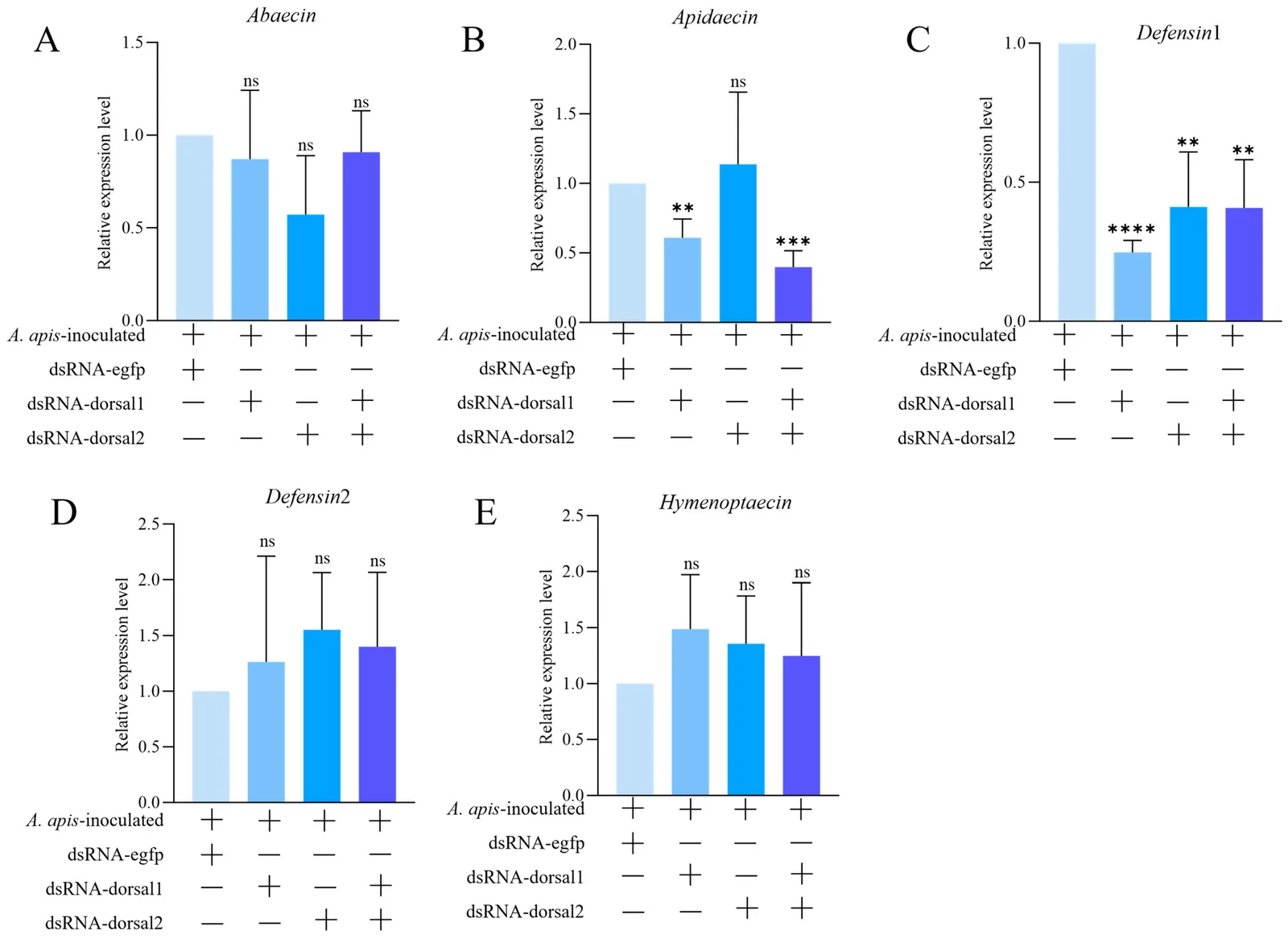
Effects of *dorsal1* and *dorsal2* knockdown on AMP gene expression in *A. apis*-inoculated larvae. Relative expression levels of *abaecin* (**A**), *apidaecin* (**B**), *defensin1* (**C**), *defensin2* (**D**), and *hymenoptaecin* (**E**) were measured in 6-day-old *A. apis*-inoculated larvae after feeding dsRNA-egfp, dsRNA-dorsal1, dsRNA-dorsal2, or dsRNA-dorsal1 plus dsRNA-dorsal2. The first bar in each panel represents the *A. apis* + dsRNA-egfp control. Data are shown as mean ± SD (*n* = 3). Statistical significance was analyzed by Student’s *t*-test, with each treatment group compared with the control group. ns, *p* > 0.05; **, *p* < 0.01, ***, *p* < 0.001, ****, *p* < 0.0001.

To examine whether reducing *dorsal1* or *dorsal2* was accompanied by changes in AMP transcripts during infection, AMP expression was measured after *dorsal* knockdown in *A. apis*-inoculated larvae. Knockdown of *dorsal1*, *dorsal2*, or both genes did not significantly alter *abaecin*, *defensin2*, or *hymenoptaecin* expression (all *p* > 0.05; Figure 5A,D,E). In contrast, *apidaecin* expression was reduced by dsRNA-*dorsal1* and by the combined dsRNA-*dorsal1* plus dsRNA-*dorsal2* treatment, with relative expression levels of 0.608 and 0.397, corresponding to 39.2% and 60.3% reductions, respectively (*p* = 0.0076 and *p* = 0.0009; Figure 5B). *defensin1* expression was reduced by dsRNA-*dorsal1*, dsRNA-*dorsal2*, and the combined treatment, with relative expression levels of 0.248, 0.411, and 0.408, corresponding to 75.2%, 58.9%, and 59.2% reductions, respectively (*p* < 0.0001, *p* = 0.0067, and *p* = 0.0041; Figure 5C). Under the tested conditions, these transcript-level associations were selective for *apidaecin* and *defensin1* rather than uniform across all five AMP genes.

### 3.3. Effects of dorsal1 Transcript Knockdown on AMP Gene Expression After A. apis Infection

**Figure 6:**
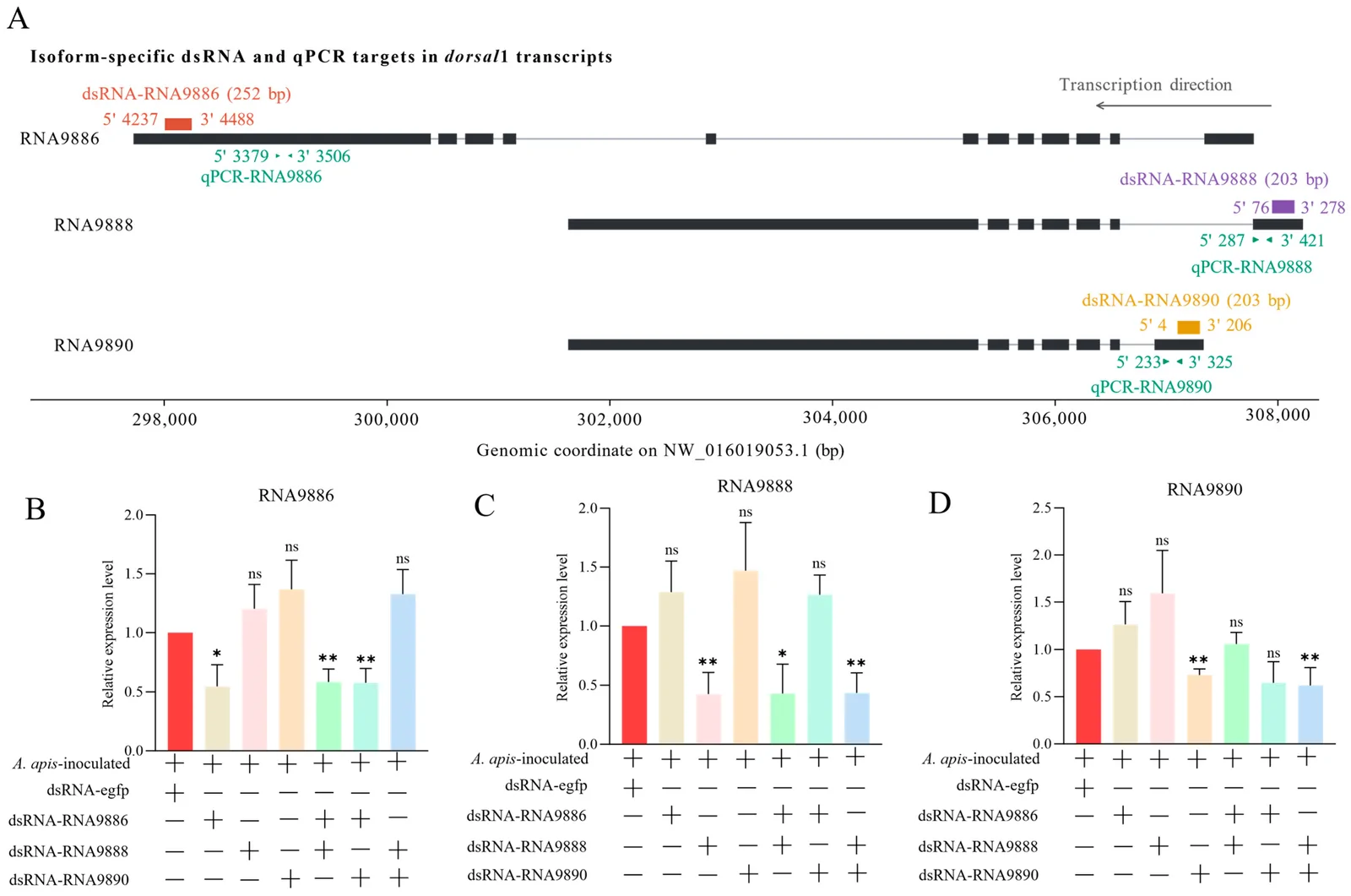
Isoform-specific knockdown of *dorsal1* transcripts in *A. apis*-inoculated larvae. (**A**) Locations of dsRNA target regions and RT-qPCR primer regions for RNA9886, RNA9888, and RNA9890. (**B**–**D**) Relative expression levels of RNA9886 (**B**), RNA9888 (**C**), and RNA9890 (**D**) in 6-day-old *A. apis*-inoculated larvae after feeding dsRNA-egfp, single transcript-targeting dsRNAs, or paired transcript-targeting dsRNAs. Data are shown as mean ± SD (*n* = 3). Statistical significance was analyzed by Student’s *t*-test, with each treatment group compared with the *A. apis* + dsRNA-egfp group. ns, *p* > 0.05; *, *p* < 0.05; **, *p* < 0.01.

Because *dorsal1* produced multiple transcript isoforms, we next examined whether targeting individual *dorsal1* transcripts was associated with distinct AMP transcript responses during *A. apis* infection. Isoform-specific dsRNA and RT-qPCR target sites were designed for RNA9886, RNA9888, and RNA9890 (Figure 6A). dsRNA-RNA9886 reduced RNA9886 expression to 0.547 of the control, corresponding to 45.3% knockdown (*p* = 0.0129), without significantly reducing RNA9888 or RNA9890 expression (*p* > 0.05; Figure 6B–D). dsRNA-RNA9888 reduced RNA9888 expression to 0.426 of the control, corresponding to 57.4% knockdown (*p* = 0.0057), without reducing RNA9886 or RNA9890 expression. dsRNA-RNA9890 reduced RNA9890 expression to 0.730 of the control, corresponding to 27.0% knockdown (*p* = 0.0020), without reducing RNA9886 or RNA9888 expression.

The paired transcript-targeting treatments showed the same target-associated pattern. dsRNA-RNA9886 plus dsRNA-RNA9888 reduced RNA9886 and RNA9888 by 41.5% and 57.0%, respectively (*p* = 0.0028 and *p* = 0.0167). dsRNA-RNA9886 plus dsRNA-RNA9890 reduced RNA9886 by 42.3% (*p* = 0.0039) and lowered RNA9890 by 35.4%, although the RNA9890 reduction was marginal (*p* = 0.0534). dsRNA-RNA9888 plus dsRNA-RNA9890 reduced RNA9888 and RNA9890 by 56.4% and 38.1%, respectively (*p* = 0.0045 and *p* = 0.0256; Figure 6B–D). These data indicate that, at the RNA level and under the tested conditions, the designed dsRNAs preferentially reduced their intended *dorsal1* transcript targets.

**Figure 7:**
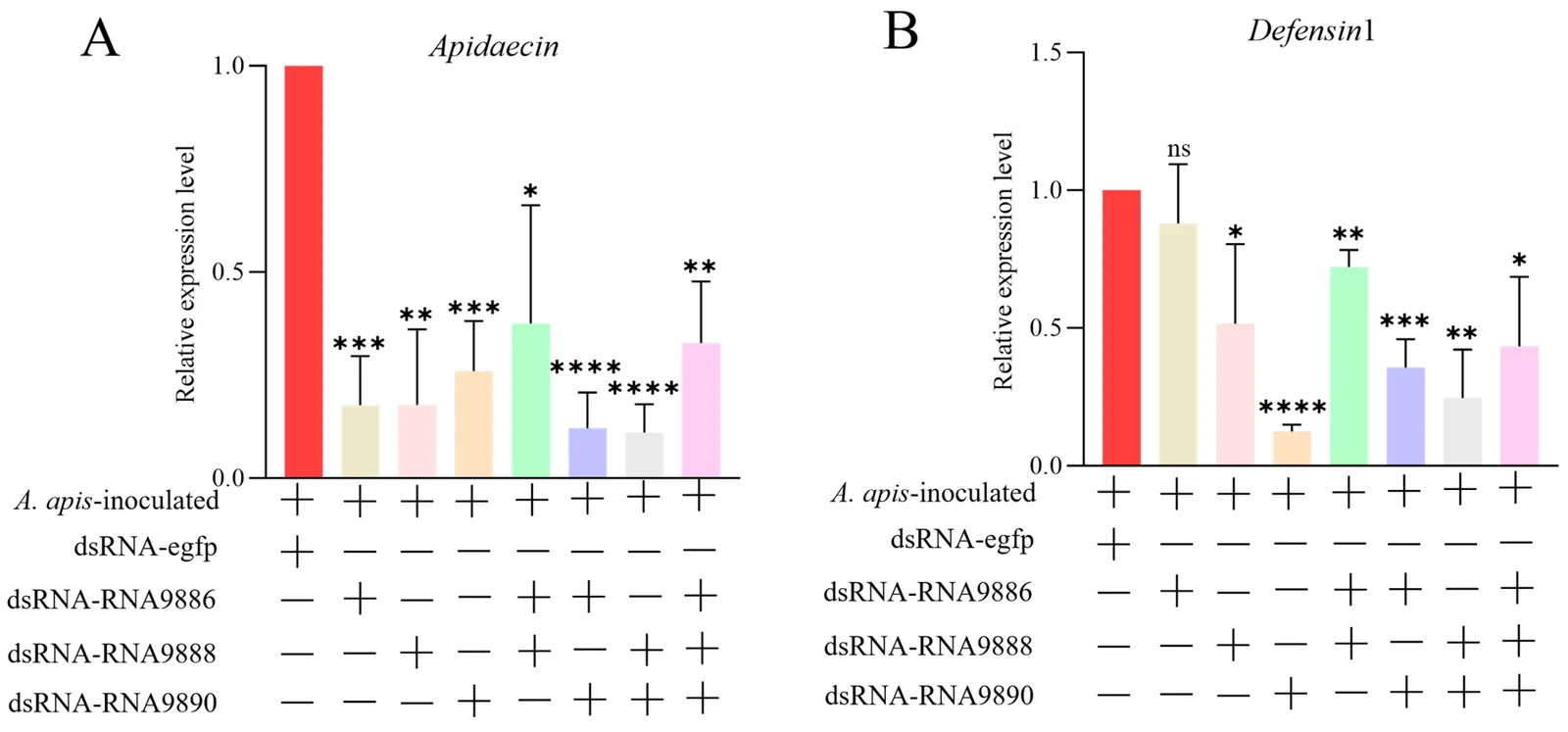
Effects of *dorsal1* transcript knockdown on *apidaecin* and *defensin1* expression in *A. apis*-inoculated larvae. Relative expression levels of *apidaecin* (**A**) and *defensin1* (**B**) were measured in 6-day-old *A. apis*-inoculated larvae after feeding dsRNA-egfp, dsRNA-RNA9886, dsRNA-RNA9888, dsRNA-RNA9890, paired transcript-targeting dsRNAs, or the combined dsRNA-RNA9886 + dsRNA-RNA9888 + dsRNA-RNA9890 treatment. The first bar in each panel represents the *A. apis* + dsRNA-egfp control. Data are shown as mean ± SD (*n* = 3). Statistical significance was analyzed by Student’s *t*-test, with each treatment group compared with the control group. ns, *p* > 0.05; *, *p* < 0.05; **, *p* < 0.01, ***, *p* < 0.001, ****, *p* < 0.0001.

We then examined AMP transcript abundance after the *dorsal1* transcript-targeting treatments in *A. apis*-inoculated larvae. *apidaecin* expression was reduced by all single, double, and triple transcript-targeting dsRNA treatments (*p* = 0.0003 for dsRNA-RNA9886, *p* = 0.0015 for dsRNA-RNA9888, *p* = 0.0005 for dsRNA-RNA9890, *p* = 0.0195 for dsRNA-RNA9886 plus dsRNA-RNA9888, *p* < 0.0001 for dsRNA-RNA9886 plus dsRNA-RNA9890, *p* < 0.0001 for dsRNA-RNA9888 plus dsRNA-RNA9890, and *p* = 0.0015 for the triple-dsRNA treatment; Figure 7A). *defensin1* expression was not significantly changed by dsRNA-RNA9886 alone (*p* = 0.3872), but was reduced by dsRNA-RNA9888 and dsRNA-RNA9890 (*p* = 0.0437 and *p* < 0.0001; Figure 7B). Double and triple transcript-targeting treatments also reduced *defensin1* expression (*p* = 0.0014, *p* = 0.0004, *p* = 0.0018, and *p* = 0.0177; Figure 7B). These results identify distinct transcript-level associations: RNA9890 knockdown was accompanied by the largest observed *defensin1* decrease, whereas knockdown of each tested transcript was accompanied by reduced *apidaecin*.

## 4. Discussion

Inoculation with *A. apis* increased the transcript abundance of *dorsal1*, *dorsal2*, and all five AMP genes examined in the midguts of 6-day-old *Apis cerana* larvae, indicating broad activation of the antifungal immune response. RNAi analysis, however, revealed a more restricted pattern of dorsal-dependent regulation. Silencing *dorsal1* reduced *apidaecin* and *defensin1* expression, whereas silencing *dorsal2* reduced *defensin1* but had no significant effect on apidaecin. Neither individual nor combined knockdown of the two Dorsal homologs significantly altered *abaecin*, *defensin2*, or *hymenoptaecin* expression. Thus, infection-induced AMP expression does not necessarily imply direct dependence on *dorsal*, and the two homologs appear to account for only part of the AMP response. This pattern is consistent with the established role of the Toll–Cactus–Dorsal module in insect antifungal immunity, while also reflecting the considerable variation in Toll pathway outputs among insect lineages, pathogens, and tissues [13,15,16,17,18,19,20]. Transcriptomic and non-coding RNA studies of *A. cerana* larvae infected with *A. apis* have likewise revealed extensive remodeling of immune and regulatory networks, supporting a multilayered response rather than control by a single transcription factor [4,32,33].

Gene-level knockdown further revealed overlapping but distinct transcript-level associations for *dorsal1* and *dorsal2*. The decrease in *defensin1* after silencing either homolog agrees with RNAi evidence from *A. mellifera* white-eyed pupae showing that Dorsal homologs participate in *defensin-1* expression [21]. The present larval-midgut infection model extends that comparison by identifying an additional association between *dorsal1* knockdown and *apidaecin*, whereas *dorsal2* knockdown did not significantly alter *apidaecin*. The absence of detectable changes in *abaecin*, *defensin2*, and *hymenoptaecin* further shows that the measured Dorsal transcripts were not uniformly associated with all *A. apis*-responsive AMP genes. Their induction may instead involve other transcription factors, immune-pathway cross-talk, or differences in the timing and cellular origin of AMP expression. Honey bees retain core Toll and IMD modules but have a reduced and reorganized immune-gene repertoire relative to *Drosophila*, and individual AMP genes cannot always be assigned exclusively to the canonical pathway relationships established in flies [16,39,40]. Because the present study targeted *dorsal* transcripts rather than upstream Toll components or the IMD-associated transcription factor Relish, it does not resolve the relative contribution of each pathway to a given AMP transcript.

Isoform-targeted RNAi further identified transcript-associated heterogeneity among the three tested *dorsal1* isoforms. Targeting RNA9886, RNA9888, or RNA9890 was accompanied by reduced *apidaecin*, whereas *defensin1* decreased after targeting RNA9888 or RNA9890 but not RNA9886 alone. Based on the transcript models underlying Figure 2, RNA9886 is predicted to encode a 587-amino-acid (aa) protein, whereas RNA9888 and RNA9890 share an identical coding sequence and encode the same 810-aa protein. All three predicted products retain the N-terminal Dorsal/Dif Rel homology DNA-binding and dimerization domains, but the RNA9886 product diverges from the RNA9888/RNA9890 product downstream of residue 325 in the C-terminal region. Because the Dorsal C-terminal region can contribute to transcriptional activation and repression in *Drosophila* [41], this coding difference provides a plausible context for the observed treatment-control pattern. Basal isoform abundance, dsRNA accessibility and knockdown efficiency, and post-transcriptional regulation may also contribute to the observed pattern. Accordingly, these findings are interpreted as transcript-level associations rather than evidence of established isoform-specific protein functions. Isoform-resolved protein quantification, transcriptional-activity assays, and direct UTR-reporter experiments will be required to distinguish these possibilities.

The results showed that a 57.6% knockdown of *dorsal1* resulted in a 39.2% reduction in apidaecin expression, whereas knockdown of the individual transcripts, with efficiencies of 45.3%, 57.4%, and 27.0%, respectively, resulted in greater reductions in apidaecin expression (62.5–89.0%). Although knockdown of either *dorsal1* or its individual transcripts consistently reduced *apidaecin* expression, an apparent inconsistency was observed in that transcript-specific knockdown produced a greater reduction in *apidaecin* expression than knockdown of *dorsal1* as a whole. Based on our current understanding, we cannot fully explain this discrepancy. One possible explanation is that knockdown of dorsal1 may affect its multiple transcripts to different extents. In addition, individual transcripts may differ in their relative contributions to the regulation of *apidaecin* expression. At the same time, we cannot exclude the possibility that measurement-related experimental variation may have contributed to the observed discrepancy.

## 5. Conclusions

**Figure 8:**
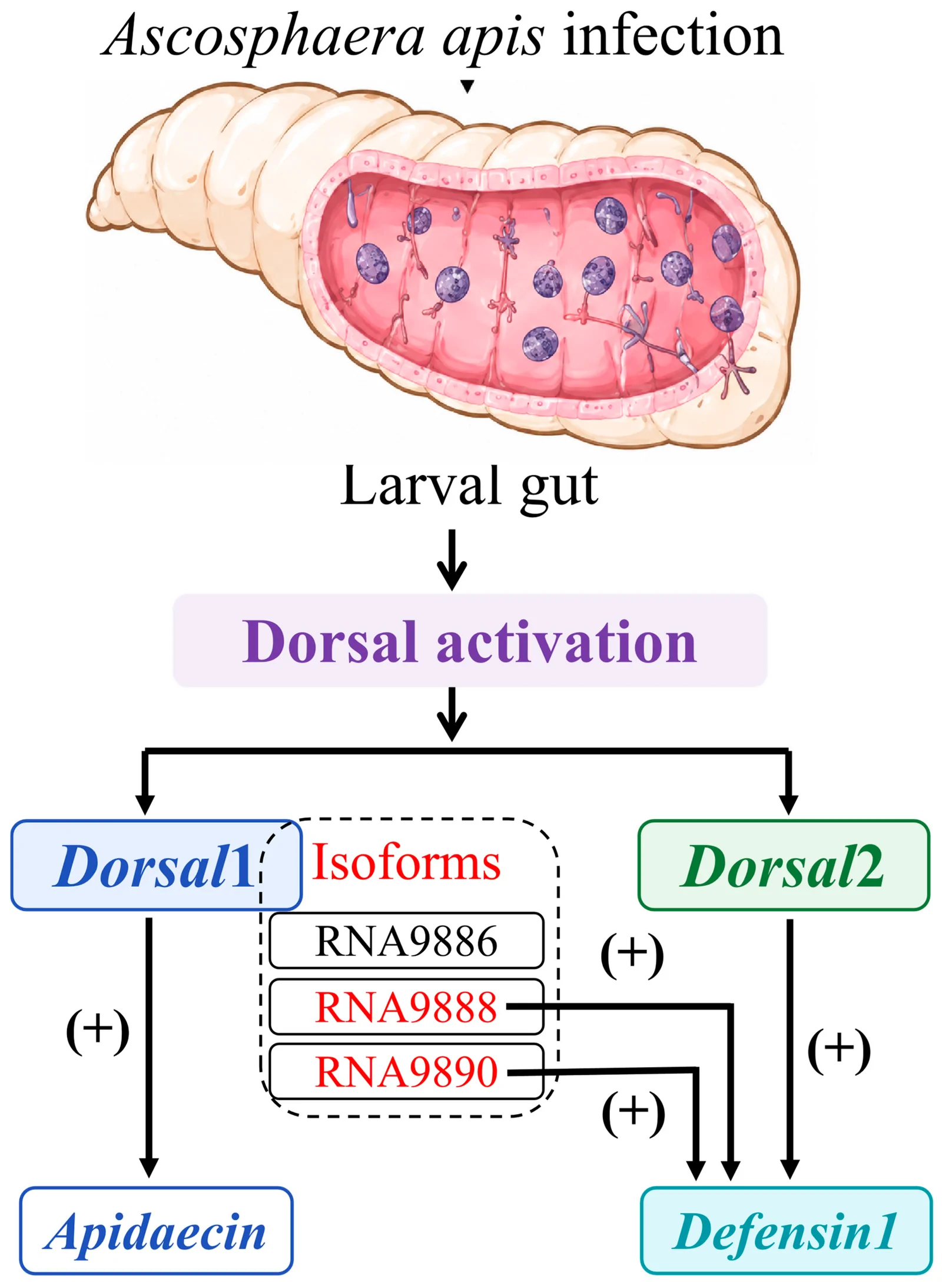
Proposed transcript-level model of Dorsal-associated antimicrobial peptide expression in *Apis cerana* larvae during *Ascosphaera apis* infection.

*A. apis* infection was associated with increased *dorsal1*, *dorsal2*, and AMP transcript abundance in the midgut of *A. cerana* larvae. RNAi-mediated reductions in *dorsal1* and *dorsal2* were accompanied by selective and partially overlapping AMP transcript changes: *dorsal1* knockdown was associated with reduced *apidaecin* and *defensin1*, whereas *dorsal2* knockdown was associated principally with reduced *defensin1* (Figure 8). Targeting selected *dorsal1* transcripts likewise produced distinct AMP transcript-response patterns (Figure 8). These data support homolog- and transcript-associated regulation at the RNA level, but they do not establish Dorsal protein abundance, nuclear localization, transcriptional activity, or direct functional differences among protein isoforms.

## Data Availability

The data supporting the conclusions of this article are included within the article and its Supplementary Materials. Additional data are available from the corresponding author upon reasonable request.

## Supplementary Materials

The following supporting information can be downloaded at: https://www.mdpi.com/article/10.3390/biom16091223/s1, Supplementary Data S1: The transcript sequences. Table S1: PCR primers used in this study.
