# Supplementary material for: Homologs and Transcriptional Isoforms of Dorsal Are Involved in the Response of Apis cerana to Ascosphaera apis Infection
Source: Biomolecules. 2026 Aug 23;16(9):1223. doi: 10.3390/biom16091223 (PMC13604300; doi:10.3390/biom16091223)
Supplement: Supplementary file 1 [file biomolecules-16-01223-s001.zip › biomolecules-4436884-supplementary.pdf]

Table S1. PCR primers used in this study.

Primer name	Primer sequence (5'-3')	Test	Amplification efficiency (%)	Mean R^2
actin-5C-F	TTATATGCCAACACTGTCCTTT	RT-qPCR	93.0 ± 4.0%	0.9995
actin-5C-R	AGAATTGATCCACCAATCCA	RT-qPCR		
dsdorsal1-F	CAGTCGCTGGTGGTATGG	Amplification of the dsRNA		
dsdorsal1-R	AAATGGTTCGCTTGTTGC	Amplification of the dsRNA		
dsdorsal2-F	CAATCCAACGCTTATTCAGT	Amplification of the dsRNA		
dsdorsal2-R	ACCATTTGTTTCTTCCACTT	Amplification of the dsRNA		
T7-*dorsal1*-F	TAATACGACTCACTATAGGGCAGTCGCTGGTGGTATGG	Vitro transcription		
T7-*dorsal1*-R	TAATACGACTCACTATAGGGAAATGGTTCGCTTGTTGC	Vitro transcription		
T7-*dorsal2*-F	TAATACGACTCACTATAGGGCAATCCAACGCTTATTCAGT	Vitro transcription		
T7-*dorsal2*-R	TAATACGACTCACTATAGGGACCATTTGTTTCTTCCACTT	Vitro transcription		
T7-*egfp*-F	TAATACGACTCACTATAGGGTACAACTACAACAGCCACAA	Vitro transcription		
T7-*egfp*-R	TAATACGACTCACTATAGGGCACCTTGATGCCGTTCTT	Vitro transcription		
dorsal1-F	AATGTGCCACTACAACCA	RT-qPCR	91.3%±11.5%	0.9994
dorsal1-R	TGCGTTACTATGACTGAGTT	RT-qPCR		
dorsal2-F	GGATCTAATGATATGCGAGC	RT-qPCR	93.1 ± 13.8%	0.9984
dorsal2-R	ACAACCTTTTCACACAACAA	RT-qPCR		
abaecin-F	GAATACGAGATGAAGGTAGT	RT-qPCR	93.5 ± 4.9%	0.9994
abaecin-R	CGTCCTGGTTGTGGTATA	RT-qPCR		
apidaecin-F	GCTGAACCTGGCAATAATC	RT-qPCR	91.6 ± 7.6%	0.9994
apidaecin-R	GTGGTCTTGGTTGTGGAA	RT-qPCR		
defensin1-F	AGCATCCTGAGAACGAAGA	RT-qPCR	92.6 ± 5.8%	0.9993
defensin1-R	CGCAAGCACTGTCATTGA	RT-qPCR		
defensin2-F	GATTCTCGTCGCTATCGT	RT-qPCR	92.5 ± 4.4%	0.9984
defensin2-R	TCAATTCCGTATCTGGTTCT	RT-qPCR		
hymenoptaecin-F	CGTGTTGGTTGTCTTCTG	RT-qPCR	91.6 ± 3.0%	0.9994
hymenoptaecin	GTTCCTTGAATGACGATGG	RT-qPCR		

-R				
dsRNA9886-F	GCAGGATTCAGTCCACAA	Amplification of the dsRNA		
dsRNA9886-R	GACATTGCGGTTACGATAC	Amplification of the dsRNA		
dsRNA9888-F	CGCTCTACAATTTCACATGA	Amplification of the dsRNA		
dsRNA9888-R	CGTATGTTCAGCGTTCTT	Amplification of the dsRNA		
dsRNA9890-F	TCTCGTCACATTGTCCTAC	Amplification of the dsRNA		
dsRNA9890-R	AATCTCTAGCAAACGAAACG	Amplification of the dsRNA		
T7-RNA9886-F	TAATACGACTCACTATAGGGGCAGGATTCAGTCCACAA	Vitro transcription		
T7-RNA9886-R	TAATACGACTCACTATAGGGGACATTGCGGTTACGATAC	Vitro transcription		
T7-RNA9888-F	TAATACGACTCACTATAGGGCGCTCTACAATTTCACATGA	Vitro transcription		
T7-RNA9888-R	TAATACGACTCACTATAGGGCGTATGTTCAGCGTTCTT	Vitro transcription		
T7-RNA9890-F	TAATACGACTCACTATAGGGTCTCGTCACATTGTCCTAC	Vitro transcription		
T7-RNA9890-R	TAATACGACTCACTATAGGGAATCTCTAGCAAACGAAACG	Vitro transcription		
RNA9886-F	GCGTTCCTTCACAGATAC	RT-qPCR	91.7 ± 2.1%	0.999
RNA9886-R	GTAACCGATACCACAGACT	RT-qPCR		
RNA9888-F	TAGTGGTTTTCAACTCAAGCT	RT-qPCR	92.0 ± 4.7%	0.9992
RNA9888-R	TCTAATGACGTCGAATTTTTT	RT-qPCR		
RNA9890-F	ATATGTGTTTGATTCACTCGT	RT-qPCR	91.6 ± 5.7%	0.9989
RNA9890-R	TATGTCGTAAAAGATGTCGTA	RT-qPCR		
EcoRI-*dorsal1*-F	GCTGATATCGGATCCGAATTCATAGTTGGATATAAAGGTCGAGCTCTAG	Prokaryotic expression vector construction.		
HindIII-*dorsal1*-R	CTCGAGTGCGGCCGCAAGCTTTCCTTCCCAAAGCACTTGTCC	Prokaryotic expression vector construction.		

Supplementary Data S1. the transcript sequences

ONT.3970.1

tataagatgtgtaacagaaatgtttgcgaatatttattttagctaagcaatttgtacgaatattgcatgcagattgtcttacaatata

tacacagtattttcttcatttatgagttttattgtatacaataataattcatattgagacattttgcatgcatgtcaagacatagatttc
gatacattttgatcacagtcacagcattttgtatacttaaatgaatatttttaattcgtaatattgaaagtaaagaattttagaaatca
tataaaaatattaacaatgtttaaaacttatcgaatgaaaaagtaatttaaaaaattacacagtaatatactgcgatacaatcaca
accatttaaatattaattacatgatatattcatgcaaagaaattttctttcagagaataatatttatataacatatttttttacaatgttat
gaagattaataaaatatacagctttttagtcaacctcccgcgttggtttatatatattatttaaagtatataattcctgaatagtgtta
ttagcaattcttgtaaaactatcggtcatgttattactttcttcaatattgcttggttctgcgtttattcctttacttgtttctggctagatt
taatttcttatttattagtaaatgataaatagaaatttgtattaataaatattatacataccttggtagaatctgaaattgataaccca
ctggatagattttcagaaagactaatatcaagatcggcgagttctgtcgaatcaagttgaggaagactcaaatcaagattata
ctgcgtgttgtccatacttaaaatatttgtaacatttgttgcttccatttcacctactatatgaattatttaaaaattacaatgatgtat
agtttgatataataataatgaatataaacacttatactaccagcattgttcaacgattgaaattcttcgtgatagtttgatgttacttt
gcttaacgttaattgttcttgcggctgtatttgtaacgtgtcatgtacatatggaaattgttgttgtgtatacgttctgattgatggtt
gttgtaagatatgactgcttgttgatggatattgaggtgatggttgtgattgaataagtgctggattgtataatctgtagtccattg
gcgatgtacgatctggcgatacttgtggcctgtacaacgtattaaaatttcttcagatccattgttctgcatattatctcatatatttt
ttaaatgatacaaagctttgtgttacctcaacgtttgtatagtagattgcgattgtggagaagttgttgccatcggatacaaggat
ggaaaggatcctccacttccaattggatattcgtaaggagatatcctttccgtcggctctgatttaataatattaaaattgtattga
tttaacattgcagcatctacaatcgtgataaaacaaatagaaatttattactattaaagactaattaaaataacacaagttcattat
ttacattttgttatttagtacaaacgatttctcttgaatgttatttcaatattttttttatatattatattaattttattaattttaataaatatata
atatgtttaaatcgaatattatcgataaattgaataaaaaaaaacataaaatgattaagaagcatataaaaattttagaagcataa
aaatggaaatgatcaagcaacatataaaaagcaaaagtttgaagtaaaaacgaaggattaattacaaaagatgcatgcaatt
ttatatattttcacttcaaacattattcgtgaaaaattgaatatcttttattagaataaaaaatacagtcaatatgattaacaaatatta
acaaactaaactaatatttttatggtcgtacctgaatatttttttatatataatattcttttgatctggaattgattgggagcagaataa
aaacacgtaatttttatcattaaataagtatgtatatatatatatagatcaatatattctgtatatatgtataagtcatacattatatag
atttttctttttcaaagcagtttataaaaatttgtattgccatacaaatattgcacataatttccgctcttattattgtattgctgaatatt
taaatacaatattttaatacaagagcaggatacttgtatattatttaaactgtaaaaattaagcccgcttttgtcaacgttattatcat
taacaacatacttttctcgtcgcattaatggatataaaaatgcatccattcgtgcaatcatcagtaataattacaaaaatacaattt
tctcttcaaatatacaaggccattgccatccaaaaatttaagtacaaattccaactatttatacaaaatacatttttaaaaaagtat
aaaacaagattcttcagtgcgtgaaaatatatatatatatgtatatatatatatgaatatagtatttcttaatacaatgaaaattctaa
ttcatgaaaaaatatcaatctttgcacaacggaaatttttttatcatttttttatgttaatcttctagattattaggcactattggacgaa
attcaacagacaaaatatataacaataagtataagaaatttttaaatttaaatatttccagtgtgcaaagatattagattaattatat
agttagtatgacgtgacttaagagcgctgaaacatagatatttcgataaatatatattcgtgattcataaaaatgctgtaaacatt
ctccaaatgtacaagttagtccaaatttttgggacttccaacaaatttttttagattcatcaatacaattattattattacaacaaata
gggttgataaaacccatcagaaaaatgcataacatgttgatgactcgcatcacaaagtcttgctaatttttttttcttgcatataga
ataaatgtgaacgcgattagtattacaaagaagtaaaatcagcaatttcgcataaatgtagcacaatgtacatttagaaattttg
acgttgcgacgatgctaatgatatcatttaaacccactcccggcaaagctgtagaattgaagacaatttgataaaaaaatactca
cgcatctcagcttctgcctgtacatgtctcaaaactagcgcgttttggctgttattaatcttttgtctctttcgccttaatgaatcag
gatcatctataaatgattgtatattaaccacatgacttttaaatattaatatatcaatttttttctgaagacaacaaacctataaattttg
atctaagtttttttaacctttaaaaatattatatctgaacaaatagttaagtcagtggttcaaaataagtttaagttacaaacaaataa
aaaatcttaaatataaattaattttaactataaattaagtgcttattctaagactagaaacaaagtgatgcctataaacttggaaca
ctatataaaaacttaacaatgtgtcataaatagtaaaaagcaatgcacaaaattaaagatatgtataataagtagcaacactata
aaatttgactagaagaaacacaaactttgatgataaataacaagtttcagccgattttatatttcagctatttttttatataaaagac
tatagaaataaaataacaaatttgtactctgactagaaaaaatagtactgcattggtttcagagtatattaatcaaagtagcataa
taaaaattgctacatgtacttttttttaaaaaatgaaaaaaatataaaatatatcttaagaacgcaaacacactcaagttacaaaaa
tttcatattattatcgtaaatacgtaacttcattcaatcataatgctttttgtttctgaaaattagcaattatctgctataacatcagcat
tttttctattataatatttgaaaaaaaatatactcttaaatggaagtaggctaaaaacacaataagacatattaaatctcctttttttc

agaataaatctttccatcttccacaggactataatagaaagacatttcatcaaattcagaagcagttgcgtgcggcgccatag
cagtatataatgcataatgttcagcttctgttaattccattccatatggtggtgtttcatcaccttccaatctaagtgattttacgcta
gtagtacttactacactagctcttggaatttgtaattgtgaattatttgatgtattgtttgttgtcaaatatgaaatgtttcctgtagta
gtcaaagattgagtagtttctttggatacatttaaaattgtgtctttatctttttcactttttttgcaaataattttgaaaataatccttgt
ttcgagtttttagttaaagtactaggtaaggaaggtaaagttttatttggtgcttcaatgaaagaatctggtgtagtatgacagga
tataggacgaggtaaaagaggcatagaaggcattttccggatacgtttcggtggtaaaggcggtaatctttcttccgtttcctg
cgttccatctcttttcgtagtcactggcggggaaggacaagattgattagtatctgtttttagtacacacacatcttcatactttctt
tcgttcgataaatcggtaaattcaataggatttttcatagccatttgtaaactagtataagtctgattatcgcaaacatcaatatcc
atagattgatcagttgtattctgttcaaatgctgcctgcactaactttgcatgtgtatcggcataaatttgatctaattctgctacttg
tgttaataaatcattgaaagacttattacaatcttctgtcacttcaattttaaaatctatttcattttctttctctttgagacatatttgtcc
tttttgtacccatttactcacttcagaataatcgaaccaatcagatttatcttttaatatttctgtattttttaatacgcacgcgttttca
acagtattagatttaggacgagttaaaatttctaaattcatgtccttcgtttctttatctatataattgggataagatttgtacattcta
tttaattgttcgttttctatagttcgtttctcttcattgattgataaatcattatttcggtaatctataatagtatttttagcatgatctatat
tttgtgcaatattttgatttatttccatacttccattataagaatctaatgtattttttacattgtacaaatcattcaaagcacgtaacgc
agaaattttattattccatttttttgtgtcgaagtcattattgttatattcgtcaatattacgcggaaatttcggagtaacgtttgaaaa
caatgtcggttcagtagctaaaattttgccaaatgaagtgtaatctgttttcttcctggcaaatgctttccgtaaagaccagaaa
gcaggcctacctgcaccgagaggtaacatcagaaaaggaaatggttcgcttgttgcaccatctgatggtcttttaagttgaat
atatacttgcactggttgttccacttgctgcatacgataagtcggtgttctaaatgcaattgctgtctattaagataaatcaatttat
caaattaatattaataaagatacaaaatttaaataattttatataaaaatcgaatttcatacttgtttatgtacatgaacaggttgaa
aatcaccaaatccttcccaaagcacttgtccatctttttcttcaaagaatctaacttgtatatcttcttttgcaaccttctcacataat
aaaatcatttccataccaccagcgactgatgcgttactatgactgagtttgcatatcacaagatctgacattgcctcttaaatatt
taatatttaattatttaatacataatcgaataattgatatcttgataaaatataattaatctgaacatacttttatcaaatataggatcg
gatactactggttgtagtggcacattgaattttctttttttgtgatccttctaaaaagacttgaaagcataatctcactgcattgagat
caatactagtaggttgtcttttgtgttcgaagccagctattaaaaaattaaatgtgatttgttttgtgccaaatatagttaaataaat
ctactgaattttaatttacttcgaaaaggatctacacgaatttcttctcttattttaagcgcctcttcaatatctttttttttacacattg
aattccaagatttgcaaatgtaactgtcatattctctgatgaaacttctactgtacaaacaccttgtttacatgcttcctttccaaca
agattatgaggatgaggtctgtatggttgatcttttgttacacatgatactacaactagagctcgacctttatatccaactatctgt
aatcagtatcagttgtatgttattaattagtattattatatattgttttttttcttcttacttttatagtaggaaatgttttattttctgatgta
ctatttacaccaggtatactaccagctgatctgccttcacattcatagcgaaaacgtaaagctttgcttgctggttgttctattattt
caacataaggcaataatcgttctgtgttcatttctcttggaatttgatcttccctcccattatatttagtatcagtttcaatgacttcaa
ctatacaaattatgaaataaaatataatgtaaaaaataaaatgtaatttatgaaatttatgtaaaaatataataaaattttcaatactt
actgacatcactcatatgtatatttccatcactcatatcatggaattgttccatgcctcttactgtttaatacattgaataatagtcat
ctgaaaaatgatttcttatcatcttatgttattaaaataatttctattttattatatttctaatattatacagaatttttttaacaattctatta
gaacaattatataaaatataaaatgattgtaaagtaacaatatatcaaaaaatttttttattcttttatattttgaacactttcatcaaa
gtgagagcaaaaattaactacatgatctaactcttgacagaaacttcataatattatgtagaaaaaatttcgcgcgcgtttagtt
aagataaatggaaatataataattggacagtaataaatacctagaaagaaataaaatgtatagaaggaaaattgattttgggat
caatttttttgatgtaaagaaaattggatgttcataaattcatgaatcgttcatcttgatagaaaaatatatttatgtcgtaaaagat
gtcgtatagatgatgattgatataaaacgtgttaggcgtaacttttgattaacactaacgagtgaatcaaacacatattaatgaa
catggataaaaatttgttaaatctctagcaaacgaaacgacagctgttcacaggtatacttctaaagacactgcacacaagtgt
cccaagtcccaagggaagagaaacgagagtagagagtgaggagggatgccatgctgatccaatcattattttgggaaagc
ctagcttcgtacaagccgtgcagctataattaaactatcgtaggacaatgtgacgagaaagacttgcctgcagcttgatcaac
atagtaaggcactttcgaatctgaaagcacgcgcactgttttggataattcttctactaggaaatgcttatattcctgaaattttttt
ataaaacacgcactgtgctaaataaattgaagaaacgcgatgtgttaatgtttagatctataattgaaattcttgtatttgtcaaat
attgctgcgcacactatttgcacgcgacataaaagcatgcacggccgtgact

ONT.3970.2

catttatgagttttattgtatacaataataattcatattgagacattttgcatgcatgtcaagacatagatttcgatacattttgatca
cagtcacagcattttgtatacttaaatgaatatttttaattcgtaatattgaaagtaaagaattttagaaatcatataaaaatattaac
aatgtttaaaacttatcgaatgaaaaagtaatttaaaaaattacacagtaatatactgcgatacaatcacaaccatttaaatatta
attacatgatatattcatgcaaagaaattttctttcagagaataatatttatataacatatttttacaatgttatgaagattaataaaa
tatacagctttttagtcaacctcccgcgttggtttatatatattatttaaagtatataattcctgaatagtgttattagcaattcttgtaa
aactatcggtcatgttattactttcttcaatattgcttggttctgcgtttattcctttacttgtttctggctagatttaatttcttatttatta
gtaaatgataaatagaaatttgtattaataaatattatacataccttggtagaatctgaaattgataacccactggatagattttca
gaaagactaatatcaagatcggcgagttctgtcgaatcaagttgaggaagactcaaatcaagattatactgcgtgttgtccat
acttaaaatatttgtaacatttgttgcttccatttcacctactatatgaattatttaaaaattacaatgatgtatagtttgatataataat
aatgaatataaacacttatactaccagcattgttcaacgattgaaattcttcgtgatagtttgatgttactttgcttaacgttaattgt
tcttgcggctgtatttgtaacgtgtcatgtacatatggaaattgttgttgtgtatacgttctgattgatggttgttgtaagatatgact
gcttgttgatggatattgaggtgatggttgtgattgaataagtgctggattgtataatctgtagtccattggcgatgtacgatctg
gcgatacttgtggcctgtacaacgtattaaaatttcttcagatccattgttctgcatattatctcatatatttttaaatgatacaaag
ctttgtgttacctcaacgtttgtatagtagattgcgattgtggagaagttgttgccatcggatacaaggatggaaaggatcctcc
acttccaattggatattcgtaaggagatatcctttccgtcggctctgatttaataatattaaaattgtattgatttaacattgcagca
tctacaatcgtgataaaacaaatagaaatttattactattaaagactaattaaaataacacaagttcattatttacattttgttattta
gtacaaacgatttctcttgaatgttatttcaatatttttttatatattatattaattttattaattttaataaatatataatatgtttaaatcga
atattatcgataaattgaataaaaaaaaacataaaatgattaagaagcatataaaaattttagaagcataaaaatggaaatgat
caagcaacatataaaaagcaaaagtttgaagtaaaaacgaaggattaattacaaaagatgcatgcaattttatatattttcactt
caaacattattcgtgaaaaattgaatatcttttattagaataaaaaatacagtcaatatgattaacaaatattaacaaactaaacta
atatttttatggtcgtacctgaatatttttttatatataatattcttttgatctggaattgattgggagcagaataaaaacacgtaattttt
atcattaaataagtatgtatatatatatatagatcaatatattctgtatatatgtataagtcatacattatatagatttttctttttcaaag
cagtttataaaaatttgtattgccatacaaatattgcacataatttccgctcttattattgtattgctgaatatttaaatacaatatttta
atacaagagcaggatacttgtatattatttaaactgtaaaaattaagcccgcttttgtcaacgttattatcattaacaacatactttt
ctcgtcgcattaatggatataaaaatgcatccattcgtgcaatcatcagtaataattacaaaaatacaattttctcttcaaatatac
aaggccattgccatccaaaaatttaagtacaaattccaactatttatacaaaatacatttttaaaaaagtataaaacaagattctt
cagtgcgtgaaaatatatatatatatgtatatatatatatgaatatagtatttcttaatacaatgaaaattctaattcatgaaaaaata
tcaatctttgcacaacggaaattttttatcatttttttatgttaatcttctagattattaggcactattggacgaaattcaacagacaa
aatatataacaataagtataagaaatttttaaatttaaatatttccagtgtgcaaagatattagattaattatatagttagtatgacgt
gacttaagagcgctgaaacatagatatttcgataaatatatattcgtgattcataaaaatgctgtaaacattctccaaatgtacaa
gttagtccaaatttttgggacttccaacaaatttttagattcatcaatacaattattattattacaacaaatagggttgataaaacc
catcagaaaaatgcataacatgttgatgactcgcatcacaaagtcttgctaatttttttttcttgcatatagaataaatgtgaacgc
gattagtattacaaagaagtaaaatcagcaatttcgcataaatgtagcacaatgtacatttagaaattttgacgttgcgacgatg
ctaatgatatcatttaaacccactcccggcaaagctgtagaattgaagacaatttgataaaaaatactcacgcatctcagcttct
gcctgtacatgtctcaaaactagcgcgttttggctgttattaatcttttgtctctttcgccttaatgaatcaggatcatctataaatg
attgtatattaaccacatgacttttaaatattaatatatcaatttttttctgaagacaacaaacctataaattttgatctaagttttttaac
ctttaaaaatattatatctgaacaaatagttaagtcagtggttcaaaataagtttaagttacaaacaaataaaaaatcttaaatata
aattaattttaactataaattaagtgcttattctaagactagaaacaaagtgatgcctataaacttggaacactatataaaaactta
acaatgtgtcataaatagtaaaaagcaatgcacaaaattaaagatatgtataataagtagcaacactataaaatttgactagaa
gaaacacaaactttgatgataaataacaagtttcagccgattttatatttcagctatttttttatataaaagactatagaaataaaat
aacaaatttgtactctgactagaaaaaatagtactgcattggtttcagagtatattaatcaaagtagcataataaaaattgctaca
tgtactttttttaaaaaatgaaaaaaatataaaatatatcttaagaacgcaaacacactcaagttacaaaaatttcatattattatcg

taaatacgtaacttcattcaatcataatgctttttgtttctgaaaattagcaattatctgctataacatcagcatttttctattataatat
ttgaaaaaaaatatactcttaaatggaagtaggctaaaaacacaataagacatattaaatctcctttttttcagaataaatctttcc
atcttccacaggactataatagaaagacatttcatcaaattcagaagcagttgcgtgcggcgccatagcagtatataatgcat
aatgttcagcttctgttaattccattccatatggtggtgtttcatcaccttccaatctaagtgattttacgctagtagtacttactaca
ctagctcttggaatttgtaattgtgaattatttgatgtattgtttgttgtcaaatatgaaatgtttcctgtagtagtcaaagattgagta
gtttctttggatacatttaaaattgtgtctttatctttttcactttttttgcaaataattttgaaaataatccttgtttcgagtttttagttaa
agtactaggtaaggaaggtaaagttttatttggtgcttcaatgaaagaatctggtgtagtatgacaggatataggacgaggta
aaagaggcatagaaggcattttccggatacgtttcggtggtaaaggcggtaatctttcttccgtttcctgcgttccatctcttttc
gtagtcactggcggggaaggacaagattgattagtatctgtttttagtacacacacatcttcatactttctttcgttcgataaatc
ggtaaattcaataggatttttcatagccatttgtaaactagtataagtctgattatcgcaaacatcaatatccatagattgatcagt
tgtattctgttcaaatgctgcctgcactaactttgcatgtgtatcggcataaatttgatctaattctgctacttgtgttaataaatcatt
gaaagacttattacaatcttctgtcacttcaattttaaaatctatttcattttctttctctttgagacatatttgtcctttttgtacccattt
actcacttcagaataatcgaaccaatcagatttatcttttaatatttctgtatttttttaatacgcacgcgttttcaacagtattagattt
aggacgagttaaaatttctaaattcatgtccttcgtttctttatctatataattgggataagatttgtacattctatttaattgttcgtttt
ctatagttcgtttctcttcattgattgataaatcattatttcggtaatctataatagtatttttagcatgatctatattttgtgcaatatttt
gatttatttccatacttccattataagaatctaatgtattttttacattgtacaaatcattcaaagcacgtaacgcagaaattttattat
tccatttttttgtgtcgaagtcattattgttatattcgtcaatattacgcggaaatttcggagtaacgtttgaaaacaatgtcggttc
agtagctaaaattttgccaaatgaagtgtaatctgttttcttcctggcaaatgctttccgtaaagaccagaaagcaggcctacct
gcaccgagaggtaacatcagaaaaggaaatggttcgcttgttgcaccatctgatggtcttttaagttgaatatatacttgcact
ggttgttccacttgctgcatacgataagtcggtgttctaaatgcaattgctgtctattaagataaatcaatttatcaaattaatatta
ataaagatacaaaatttaaataattttatataaaaatcgaatttcatacttgtttatgtacatgaacaggttgaaaatcaccaaatc
cttcccaaagcacttgtccatctttttcttcaaagaatctaacttgtatatcttcttttgcaaccttctcacataataaaatcatttcca
taccaccagcgactgatgcgttactatgactgagtttgcatatcacaagatctgacattgcctcttaaatatttaatatttaattatt
taatacataatcgaataattgatatcttgataaaatataattaatctgaacatacttttatcaaatataggatcggatactactggtt
gtagtggcacattgaattttctttttgtgatccttctaaaaagacttgaaagcataatctcactgcattgagatcaatactagtag
gttgtcttttgtgttcgaagccagctattaaaaaattaaatgtgatttgttttgtgccaaatatagttaaataaatctactgaattttaa
tttacttcgaaaaggatctacacgaatttcttctcttattttaagcgcctcttcaatatcttttttttttacacattgaattccaagatttg
caaatgtaactgtcatattctctgatgaaacttctactgtacaaacaccttgtttacatgcttcctttccaacaagattatgaggat
gaggtctgtatggttgatcttttgttacacatgatactacaactagagctcgacctttatatccaactatctgtaatcagtatcagtt
gtatgttattaattagtattattatatattgttttttttttcttcttacttttatagtaggaaatgttttattttctgatgtactatttacaccag
gtatactaccagctgatctgccttcacattcatagcgaaaacgtaaagctttgcttgctggttgttctattatttcaacataaggc
aataatcgttctgtgttcatttctcttggaatttgatcttccctcccattatatttagtatcagtttcaatgacttcaactatacaaatta
tgaaataaaatataatgtaaaaaataaaatgtaatttatgaaatttatgtaaaaatataataaaattttcaatacttactgacatcac
tcatatgtatatttccatcactcatatcatggaattgttccatgcctcttactgtttaatacattgaataatagtcatctgaaaaatga
tttcttatcatcttatgttattaaaataatttctattttattatatttctaatattatacagaattttttttaacaattctattagaacaattatat
aaaatataaaatgattgtaaagtaacaatatatcaaaaaatttttttttattcttttatattttgaacactttcatcaaagtgagagcaa
aaattaactacatgatctaactcttgacagaaacttcataatattatgtagaaaaaaatttcgcgcgcgtttagttaagataaatgg
aaatataataattggacagtaataaatacctagaaagaaataaaatgtatagaaggaaaattgattttgggatcaatttttttgat
gtaaagaaaattggatgttcataaattcatgaatcgttcatcttgatagaaaaatatatttatgtcgtaaaagatgtcgtatagatg
atgattgatataaaacgtgttaggcgtaacttttgattaacactaacgagtgaatcaaacacatattaatgaacatggataaaaa
tttgttaaatctctagcaaacgaaacgacagctgttcacaggtatacttctaaagacactgcacacaagtgtcccaagtccca
agggaagagaaacgagagtagagagtgaggagggatgccatgctgatccaatcattattttgggaaagcctagcttcgtac
aagccgtgcagctataattaaactatcgtaggacaatgtgacgagaaagacttgcctgcagcttgatcaacatagtaaggca
ctttcgaatctgaaagcacgcgcactgttttggataattcttctactaggaaatgcttatattcctgaaatttttttataaaacacgc

actgtgctaaataaattgaagaaacgcgatgtgttaatgtttagatctataattgaaattcttgtatttgtcaaatattgctgcgca
cactatttgcacgcgacataaaagcatgcacggccgtgactagagacaagactttatgcggcataacattttgctcggacttt
gtatcggtact

RNA5296
ttcaataatatcattaaaaattcatttagaaaaattattacccattattattctttttttactatcttttctttgctcttgctcttcatttaaa
ctctcgtttagctaaatttaataacaacaataataattaatttttattcatttaataaactaattaggaaatatagatatgaaatatata
ccttagaaatatccgaaatggataatatattagatagattttcagatagattttcaatattaaaattataaagttcttctgaattaattt
ttaaaagattcgaatcaaaatcgcattctaagctatccatatttatggtatttataccatttgtttcttccacttcacctgttacgtaa
attattatctcggaataagatcaaaataacagtatacatatatgcagtatacataagcgcacttataatgaatgcaaaaatattta
aattaccagcgttttccaacaaatgaaattcctcgtaacgtgattctgatgtcactttagttaacgttaagtgttgttcaggttgtat
ttgtaacatgtcatgaatatatgaaaactgttcttttggacgcaagtcgattgatacttgttctgcgtcaccgtttgttaatggatat
tgaggtgatggtcgaggtgactgaataagcgttggattgttttctattggcgatatttcttccctgtgttaacattaaaattccttta
gattcgtaattatatttttgattcattattttatattcgttacatgataattatataaaaatcaaccttatagtttcattattaaattgcgg
ttgctcagaaatgatttccaaggcgcgcaatggaaacaattcttcattttctgaacattcaaatcgaggaactatcacttccgat
gattctgataataataaggttaaaatgattattgatataacagattatataattttagcagttatttacaatcacgataaaatagata
tctattaatgaaatagaataatacaaattactttattatttattacattacatattattactttcgacaaatattttagatttttcacaaag
tcagtatttagttcaaaataaatacgaatacttataacaaagaactaactattttgtaaatgtaaatgcacttttagaaattttgaca
atggaacaatgaaattatttaaattgataagttaattatttaaattgatagaatataatacaaaaataaaatactcactttcctcagt
ttccgtttctttctgtttcaattctgtgtttacatcgttattattaatctttcgtttctttcgttttaaggaattaggattatctataaattgta
cattaaaaatattatcatatcatttttttttgaagacaaatgaacgtgcgaaattcattcactataagggtatcgataaaagaaagg
tattctcttctagtgaatatttttttttacaaagattaataaataaatctacctggactgaggggtaacattagaaaaggtaatggtt
cgctcaatgcaccatctgatggccttttcagttgaatttgtactgagactggctcctcaatttcctgcgtgtgataacttgatgtttt
gaagcgaattgctgtctgcattaattaaattaattaaaggattatcaattaatctagcgtaattttatgtaaaacttaaatattacat
acatacttgtttgtgcacatgagcagggtgaaaatcaccaaatccttcccaaactactttcccattcttctcctcaaagaatctca
cttgtatatcatcttttacaaccttttcacacaacaaaatcatatgcatgccaccagcaaccgatgcgctataatgactgagctc
gcatatcattagatccggtattatctcttaaatattcaatattaattattcaatgcgcaattaaagaattataatatcttaaaaactgg
atataaacgtgtaattaaacatacttttatcgaatataggatcggatacaattggtgttaatggcacgttgaatttccctttctgcg
ttccttctaaaaacacttggaagcatagtctcactgaatttagatcaatggaattaagatcccttttgaattgaaagccagctatt
caaacaatttttaaatatgatttgtttttatatatcgagcataatcgaacaaacggaaagtgggattttaatttacttcgaaaagga
tctacacgaatctcctctcttatttttaattctcttttgatgtcgttccttttaacgcacatgataccaagatttgcaaatgtcactgtc
atgtcttctgatgcaatttcgattgtacaaacaccttttttcacatccttcccttcctatcagattatgaggatggggtctgtatggttt
gtcttttgttacacacgatgctacagctatagcctgacctttatatccaactacctacaatcaatcttatttgttattactttatatcaa
ttttaatctaaaaattaaattaaattaattctatcacgtattattttatttttcatttcttcactacttactcttatactaggacacgttttatt
ttctaatgtactgcttgcacctggtatattaccggctaatctgccttcacattcatatcgaaaacgtaaagtttttgtgggttgctct
attatttcaacatacggtagaaattttctaaaattcattttgatctttctccaatttgctaatttcaattaatgattgaaattctttcacat
accgatacttacactcttggtataagtg

RNA9886
ggactactcaagcaatgattcgttgactgtcgtagaacgggtaacagtaatcttatatgacagtatcttgcatacaatgatcagt
gtcttattaaagacatatattcgagtttattaatggtatcatatgtaatttttgatgaattacacatttcactgattatcaacaattgaa
atatatatgctctttatgttagtgtcagtggatctttgcaaatggaatggtccatgccatcacttatttcgggagactggtagaca
agaaacgggtgaagagacattgcggttacgataccattgtaccatagctgcataataaataaaggtaacgataatttgaatgc
gagacgatgtcccacgttttaatgtacaaatattttcgaatgagatagttaaataaaacgcagttagatcagagaaaatgattc

gttctcgtaagtagtaatgggtttaacttccgaaaacaaattacagtaatagcagtattaaaataaaaaagaaaactgctatttg
tggactgaatcctgctacattgaatccaaggagatgggatggactgtatacacacttgtacgataaatccaatttattcaattgg
ttaaatgaaagaaataacaaaatttaagtatactccgtatataatgatacgtaagtcatacagcaatttattgtattgtttacagac
cataccacctccagaactctgttattctacgattttatacacaatctgctgctgtatttacacgtgttgacttcaagatagtaccttg
acgatcatgttgacaaagaactatcttaaagtacaggtgcctgagcaaaaatgagtactttagcgttcatagtatacaataata
cactgtaagcataggtccatttaattttattaatacaataacaagccccgtttgcaacatgttcaaaaaatatgaaaatggactta
cgttgtgtactttgagctagagatcacggaactcttctctgctccagtatttgacgtgattctgaaaataatttactggtgctaac
gggtcttacaatcaatcaggctgagcgcccagataccgttacatccctacatttgttaggtaagcataaaaagaagaatttaa
gaatcttaatgaatctggtaatgaaacatgtcatacgattaaacgtaaacaaaatcttaggctaagaatttggtttgtgtgttcat
aagtcttgttcaaactacttttgtacaggataccatcgcatcatcactttcgacataacagtaaccgataccacagactatatttc
tgcccattccatcagaattgaactttcattgtatttttaagtaaatacattttggaaaacaacagatgttttttattcttcacgtatct
gtgaaggaacgcgtctttccctatttaatacttgacaattaatcgacaacttatttttcgaagataatttagactaagattgttttta
cgtaccttctatgtacatataaattaagaattatgtagttatactatgtaaatataaaatagcaatttacaatagaatatgtacgtaa
tatgtgatacacaagattaaatattactggatttcaatatctatgtttatttacacgtataattgtatgtaaaattataatgtattcttag
atgtaactgtatataaagtaaaaatatgtacttaagcataatattatttgtatctgatattgtttatttataaaataatttctgtaattaat
agtgattttaagataatgatactggataccaggtaaattgtaattgatgatgaacaagatattacaaatatgtgtaaaatctatga
tggaatgcaaccatagtacataatattaaaatttaaggtaaaaatttttttaaatgtgaatcaacgtacctggacggaatatatatt
attggaaatttttaattggatacatactattagaggtatagcattcagatattacccctaatataaaaataatataccatttaaagg
aatttgtagtaaatatataaatgtgtaaaaaataaaaatcaaacatttgtataagagtataagatgtgtaacagaaatgtttgcga
atatttattttagctaagcaatttgtacgaatattgcatgcagattgtcttacaatatatacacagtattttcttcatttatgagttttatt
gtatacaataataattcatattgagacattttgcatgcatgtcaagacatagatttcgatacattttgatcacagtcacagcatttt
gtatacttaaatgaatatttttaattcgtaatattgaaagtaaagaattttagaaatcatataaaaatattaacaatgtttaaaacttat
cgaatgaaaaagtaatttaaaaaattacacagtaatatactgcgatacaatcacaaccatttaaatattaattacatgatatattc
atgcaaagaaattttctttcagagaataatatttatataacatatttttttacaatgttatgaagattaataaaatatacagctttttagt
caacctcccgcgttggtttatatatattatttaaagtatataattcctgaatagtgttattagcaattcttgtaaaactatcggtcatg
ttattactttcttcaatattgcttggttctgcgtttattcctttacttgtttctggctagatttaatttcttatttattagtaaatgataaata
gaaatttgtattaataaatattatacataccttggtagaatctgaaattgataacccactggatagattttcagaaagactaatatc
aagatcggcgagttctgtcgaatcaagttgaggaagactcaaatcaagattatactgcgtgttgtccatacttaaaatatttgta
acatttgttgcttccatttcacctactatatgaattatttaaaaattacaatgatgtatagtttgatataataataatgaatataaacac
ttatactaccagcattgttcaacgattgaaattcttcgtgatagtttgatgttactttgcttaacgttaattgttcttgcggctgtattt
gtaacgtgtcatgtacatatggaaattgttgttgtgtatacgttctgattgatggttgttgtaagatatgactgcttgttgatggata
ttgaggtgatggttgtgattgaataagtgctggattgtataatctgtagtccattggcgatgtacgatctggcgatacttgtggc
ctgtacaacgtattaaaatttcttcagatccattgttctgcatattatctcatatattttttaaatgatacaaagctttgtgttacctcaa
cgtttgtatagtagattgcgattgtggagaagttgttgccatcggatacaaggatggaaaggatcctccacttccaattggata
ttcgtaaggagatatcctttccgtcggctctgatttaataatattaaaattgtattgatttaacattgcagcatctacaatcgtgata
aaacaaatagaaatttattactattaaagactaattaaaataacacaagttcattatttacattttgttatttagtacaaacgatttct
cttgaatgttatttcaatattttttttatatattatattaattttattaattttaataaatatataatatgtttaaatcgaatattatcgataaatt
gaataaaaaaaaacataaaatgattaagaagcatataaaaattttagaagcataaaaatggaaatgatcaagcaacatataaa
aagcaaaagtttgaagtaaaaacgaaggattaattacaaaagatgcatgcaattttatatattttcacttcaaacattattcgtga
aaaattgaatatcttttattagaataaaaaatacagtcaatatgattaacaaatattaacaaactaaactaatatttttatggtcgta
cctgaatatttttatatataatattcttttgatctggaattgattgggagcagaataaaaacacgtaatttttatcattaaataagtat
gtatatatatatatagatcaatatattctgtatatatgtataagtcatacattatatagatttttctttttcaaagcagtttataaaaattt
gtattgccatacaaatattgcacataatttccgctcttattattgtattgctgaatatttaaatacaatattttaatacaagagcagg
atacttgtatattatttaaactgtaaaaattaagcccgcttttgtcaacgttattatcattaacaacatacttttctcgtcgcattaatg

gatataaaaatgcatccattcgtgcaatcatcagtaataattacaaaaatacaattttctcttcaaatatacaaggccattgccat
ccaaaaatttaagtacaaattccaactatttatacaaaatacatttttaaaaaagtataaaacaagattcttcagtgcgtgaaaat
atatatatatatgtatatatatatatgaatatagtatttcttaatacaatgaaaattctaattcatgaaaaaatatcaatctttgcacaa
cggaaatttttatcatttttttatgttaatcttctagattattaggcactattggacgaaattcaacagacaaaatatataacaataa
gtataagaaatttttaaatttaaatatttccagtgtgcaaagatattagattaattatatagttagtatgacgtgacttaagagcgct
gaaacatagatatttcgataaatatatattcgtgattcataaaaatgctgtaaacattctccaaatgtacaagttagtccaaattttt
gggacttccaacaaattttttagattcatcaatacaattattattattacaacaaatagggttgataaaacccatcagaaaaatgc
ataacatgttgatgactcgcatcacaaagtcttgctaattttttttcttgcatatagaataaatgtgaacgcgattagtattacaaa
gaagtaaaatcagcaatttcgcataaatgtagcacaatgtacatttagaaattttgacgttgcgacgatgctaatgatatcattta
aacccactcccggcaaagctgtagaattgaagacaatttgataaaaaatactcacgcatctcagcttctgcctgtacatgtctc
aaaactagcgcgttttggctgttattaatcttttgtctctttcgccttaatgaatcaggatcatctataaatgattgtatattaaccac
atgacttttaaatattaatatatcaatttttctgaagacaacaaacctataaattttgatctaagtttttaacctttaaaaatattatat
ctgaacaaatagttaagtcagtggttcaaaataagtttaagttacaaacaaataaaaaatcttaaatataaattaattttaactata
aattaagtgcttattctaagactagaaacaaagtgatgcctataaacttggaacactatataaaaacttaacaatgtgtcataaa
tagtaaaaagcaatgcacaaaattaaagatatgtataataagtagcaacactataaaatttgactagaagaaacacaaactttg
atgataaataacaagtttcagccgattttatatttcagctatttttttatataaaagactatagaaataaaataacaaatttgtactct
gactagaaaaaatagtactgcattggtttcagagtatattaatcaaagtagcataataaaaattgctacatgtactttttttaaaaa
atgaaaaaaatataaaatatatcttaagaacgcaaacacactcaagttacaaaaatttcatattattatcgtaaatacgtaacttc
attcaatcataatgctttttgtttctgaaaattagcaattatctgctataacatcagcatttttctattataatatttgaaaaaaaatat
actcttaaatggaagtaggctaaaaacacaataagacatattaaatctcctttttttcagaataaatctttccatcttccacagga
ctataatagaaagacatttcatcaaattcagaagcagttgcgtgcggcgccatagcagtatataatgcataatgttcagcttct
gttaattccattccatatggtggtgtttcatcaccttccaatctaagtgattttacgctagtagtacttactacactagctcttggaa
tttgtaattgtgaattatttgatgtattgtttgttgtcaaatatgaaatgtttcctgtagtagtcaaagattgagtagtttctttggatac
atttaaaattgtgtctttatctttttcactttttttgcaaataattttgaaaataatccttgtttcgagtttttagttaaagtactaggtaa
ggaaggtaaagttttatttggtgcttcaatgaaagaatctggtgtagtatgacaggatataggacgaggtaaaagaggcata
gaaggcattttccggatacgtttcggtggtaaaggcggtaatctttcttccgtttcctgcgttccatctcttttcgtagtcactggc
ggggaaggacaagattgattagtatctgtttttagtacacacacatcttcatactttctttcgttcgataaatcggtaaattcaata
ggatttttcatagccatttgtaaactagtataagtctgattatcgcaaacatcaatatccatagattgatcagttgtattctgttcaa
atgctgcctgcactaactttgcatgtgtatcggcataaatttgatctaattctgctacttgtgttaataaatcattgaaagacttatt
acaatcttctgtcacttcaattttaaaatctatttcattttctttctctttgagacatatttgtcctttttgtacccatttactcacttcaga
ataatcgaaccaatcagatttatcttttaatatttctgtatttttttaatacgcacgcgttttcaacagtattagatttaggacgagtta
aaatttctaaattcatgtccttcgtttctttatctatataattgggataagatttgtacattctatttaattgttcgttttctatagttcgttt
ctcttcattgattgataaatcattatttcggtaatctataatagtatttttagcatgatctatattttgtgcaatattttgatttatttccat
acttccattataagaatctaatgtatttttttacattgtacaaatcattcaaagcacgtaacgcagaaattttattattccatttttttgtg
tcgaagtcattattgttatattcgtcaatattacgcggaaatttcggagtaacgtttgaaaacaatgtcggttcagtagctaaaatt
ttgccaaatgaagtgtaatctgttttcttcctggcaaatgctttccgtaaagaccagaaagcaggcctacctgcaccgagagg
taacatcagaaaaggaaatggttcgcttgttgcaccatctgatggtcttttaagttgaatatatacttgcactggttgttccacttg
ctgcatacgataagtcggtgttctaaatgcaattgctgtctattaagataaatcaatttatcaaattaatattaataaagatacaaa
atttaaataattttatataaaaatcgaatttcatacttgtttatgtacatgaacaggttgaaaatcaccaaatccttcccaaagcact
tgtccatctttttcttcaaagaatctaacttgtatatcttcttttgcaaccttctcacataataaaatcatttccataccaccagcgac
tgatgcgttactatgactgagtttgcatatcacaagatctgacattgcctcttaaatatttaatatttaattatttaatacataatcga
ataattgatatcttgataaaatataattaatctgaacatacttttatcaaatataggatcggatactactggttgtagtggcacattg
aattttcttttttgtgatccttctaaaaagacttgaaagcataatctcactgcattgagatcaatactagtaggttgtcttttgtgttc
gaagccagctattaaaaaattaaatgtgatttgttttgtgccaaatatagttaaataaatctactgaattttaatttacttcgaaaag

gatctacacgaatttcttctcttattttaagcgcctcttcaatatcttttttttttacacattgaattccaagatttgcaaatgtaactgt
catattctctgatgaaacttctactgtacaaacaccttgtttacatgcttcctttccaacaagattatgaggatgaggtctgtatgg
ttgatcttttgttacacatgatactacaactagagctcgacctttatatccaactatctgtaatcagtatcagttgtatgttattaatta
gtattattatatattgtttttttttcttcttacttttatagtaggaaatgttttattttctgatgtactatttacaccaggtatactaccagct
gatctgccttcacattcatagcgaaaacgtaaagctttgcttgctggttgttctattatttcaacataaggcaataatcgttctgtg
ttcatttctcttggaatttgatcttccctcccattatatttagtatcagtttcaatgacttcaactatacaaattatgaaataaaatata
atgtaaaaaataaaatgtaatttatgaaatttatgtaaaaatataataaaattttcaatacttactgacatcactcatatgtatatttc
catcactcatatcatggaattgttccatgcctcttactgtttaatacattgaataatagtcatctgaaaaatgatttcttatcatcttat
gttattaaaataatttctattttattatatttctaatattatacagaatttttttaacaattctattagaacaattatataaaatataaaatg
attgtaaagtaacaatatatcaaaaaatttttttttattcttttatattttgaacactttcatcaaagtgagagcaaaaattaactacatg
atctaactcttgacagaaacttcataatattatgtagaaaaaatttcgcgcgcgtttagttaagataaatggaaatataataattg
gacagtaataaatacctagaaagaaataaaatgtatagaaggaaaattgattttgggatcaatttttttgatgtaaagaaaattg
gatgttcataaattcatgaatcgttcatcttgatagaaaaatatatttatgtcgtaaaagatgtcgtatagatgatgattgatataaa
acgtgttaggcgtaacttttgattaacactaacgagtgaatcaaacacatattaatgaacatggataaaaatttgttaaatctcta
gcaaacgaaacgacagctgttcacaggtatacttctaaagacactgcacacaagtgtcccaagtcccaagggaagagaaa
cgagagtagagagtgaggagggatgccatgctgatccaatcattattttgggaaagcctagcttcgtacaagccgtgcagc
tataattaaactatcgtaggacaatgtgacgagaaagacttgcctgcagcttgatcaacatagtaaggcactttcgaatctgaa
agcacgcgcactgttttggataattcttctactaggaaatgcttatattcctgaaatttttttataaaacacgcactgtgctaaata
aattgaagaaacgcgatgtgttaatgtttagatctataattgaaattcttgtatttgtcaaatattgctgcgcacactatttgcacg
cgacataaaagcatgcacggccgtgactagagacaagactttatgcggcataacattttgctcggactttgtatcggtactga
catgtccgttgcttttaatggtagaagcaaagcaatatgctatatacttcgcggatacgcgcaagtacgcgtaccctgacggtt
ctatactcacaaccaattcaaagtgagtgaacgtgtgttaacattatatactacctttcgatt

RNA9888
attaacaaactaaactaatatttttatggtcgtacctgaatatttttttatatataatattcttttgatctggaattgattgggagcagaa
taaaaacacgtaatttttatcattaaataagtatgtatatatatatatagatcaatatattctgtatatatgtataagtcatacattatat
agatttttctttttcaaagcagtttataaaaatttgtattgccatacaaatattgcacataatttccgctcttattattgtattgctgaat
atttaaatacaatattttaatacaagagcaggatacttgtatattatttaaactgtaaaaattaagcccgcttttgtcaacgttattat
cattaacaacatacttttctcgtcgcattaatggatataaaaatgcatccattcgtgcaatcatcagtaataattacaaaaataca
attttctcttcaaatatacaaggccattgccatccaaaaatttaagtacaaattccaactatttatacaaaatacatttttaaaaaag
tataaaacaagattcttcagtgcgtgaaaatatatatatatatgtatatatatatatgaatatagtatttcttaatacaatgaaaattc
taattcatgaaaaaatatcaatctttgcacaacggaaatttttttatcatttttttatgttaatcttctagattattaggcactattggac
gaaattcaacagacaaaatatatataacaataagtataagaaatttttaaatttaaatatttccagtgtgcaaagatattagattaatt
atatagttagtatgacgtgacttaagagcgctgaaacatagatatttcgataaatatatattcgtgattcataaaaatgctgtaaa
cattctccaaatgtacaagttagtccaaatttttgggacttccaacaaatttttttagattcatcaatacaattattattattacaacaa
atagggttgataaaacccatcagaaaaatgcataacatgttgatgactcgcatcacaaagtcttgctaatttttttttcttgcatat
agaataaatgtgaacgcgattagtattacaaagaagtaaaatcagcaatttcgcataaatgtagcacaatgtacatttagaaat
tttgacgttgcgacgatgctaatgatatcatttaaacccactcccggcaaagctgtagaattgaagacaatttgataaaaaata
ctcacgcatctcagcttctgcctgtacatgtctcaaaactagcgcgttttggctgttattaatcttttgtctctttcgccttaatgaat
caggatcatctataaatgattgtatattaaccacatgacttttaaatattaatatatcaattttttctgaagacaacaaacctataaat
tttgatctaagtttttttaacctttaaaaatattatatctgaacaaatagttaagtcagtggttcaaaataagtttaagttacaaacaaa
taaaaaatcttaaatataaattaattttaactataaattaagtgcttattctaagactagaaacaaagtgatgcctataaacttgga
acactatataaaaacttaacaatgtgtcataaatagtaaaaagcaatgcacaaaattaaagatatgtataataagtagcaacac
tataaaatttgactagaagaaacacaaactttgatgataaataacaagtttcagccgattttatatttcagctatttttttatataaaa

gactatagaaataaaataacaaatttgtactctgactagaaaaaatagtactgcattggtttcagagtatattaatcaaagtagc
ataataaaaattgctacatgtactttttttaaaaaatgaaaaaaatataaaatatatcttaagaacgcaaacacactcaagttaca
aaaatttcatattattatcgtaaatacgtaacttcattcaatcataatgctttttgtttctgaaaattagcaattatctgctataacatc
agcatttttctattataatatttgaaaaaaaatatactcttaaatggaagtaggctaaaaacacaataagacatattaaatctcctt
tttttcagaataaatctttccatcttccacaggactataatagaaagacatttcatcaaattcagaagcagttgcgtgcggcgcc
atagcagtatataatgcataatgttcagcttctgttaattccattccatatggtggtgtttcatcaccttccaatctaagtgattttac
gctagtagtacttactacactagctcttggaatttgtaattgtgaattatttgatgtattgtttgttgtcaaatatgaaatgtttcctgt
agtagtcaaagattgagtagtttctttggatacatttaaaattgtgtctttatctttttcacttttttgcaaataattttgaaaataatc
cttgtttcgagtttttagttaaagtactaggtaaggaaggtaaagttttatttggtgcttcaatgaaagaatctggtgtagtatgac
aggatataggacgaggtaaaagaggcatagaaggcattttccggatacgtttcggtggtaaaggcggtaatctttcttccgtt
tcctgcgttccatctcttttcgtagtcactggcggggaaggacaagattgattagtatctgtttttagtacacacacatcttcatac
tttctttcgttcgataaatcggtaaattcaataggatttttcatagccatttgtaaactagtataagtctgattatcgcaaacatcaat
atccatagattgatcagttgtattctgttcaaatgctgcctgcactaactttgcatgtgtatcggcataaatttgatctaattctgct
acttgtgttaataaatcattgaaagacttattacaatcttctgtcacttcaattttaaaatctatttcattttctttctctttgagacatatt
tgtcctttttgtacccatttactcacttcagaataatcgaaccaatcagatttatcttttaatatttctgtattttttaatacgcacgcgt
tttcaacagtattagatttaggacgagttaaaatttctaaattcatgtccttcgtttctttatctatataattgggataagatttgtaca
ttctatttaattgttcgttttctatagttcgtttctcttcattgattgataaatcattatttcggtaatctataatagtatttttagcatgatc
tatattttgtgcaatattttgatttatttccatacttccattataagaatctaatgtattttttacattgtacaaatcattcaaagcacgta
acgcagaaattttattattccatttttttgtgtcgaagtcattattgttatattcgtcaatattacgcggaaatttcggagtaacgtttg
aaaacaatgtcggttcagtagctaaaattttgccaaatgaagtgtaatctgttttcttcctggcaaatgctttccgtaaagaccag
aaagcaggcctacctgcaccgagaggtaacatcagaaaaggaaatggttcgcttgttgcaccatctgatggtcttttaagttg
aatatatacttgcactggttgttccacttgctgcatacgataagtcggtgttctaaatgcaattgctgtctattaagataaatcaatt
tatcaaattaatattaataaagatacaaaatttaaataattttatataaaaatcgaatttcatacttgtttatgtacatgaacaggttg
aaaatcaccaaatccttcccaaagcacttgtccatctttttcttcaaagaatctaacttgtatatcttcttttgcaaccttctcacata
ataaaatcatttccataccaccagcgactgatgcgttactatgactgagtttgcatatcacaagatctgacattgcctcttaaata
tttaatatttaattatttaatacataatcgaataattgatatcttgataaaatataattaatctgaacatacttttatcaaatataggatc
ggatactactggttgtagtggcacattgaattttctttttttgtgatccttctaaaaagacttgaaagcataatctcactgcattgag
atcaatactagtaggttgtcttttgtgttcgaagccagctattaaaaaattaaatgtgatttgttttgtgccaaatatagttaaataa
atctactgaattttaatttacttcgaaaaggatctacacgaatttcttctcttattttaagcgcctcttcaatatcttttttttttacacatt
gaattccaagatttgcaaatgtaactgtcatattctctgatgaaacttctactgtacaaacaccttgtttacatgcttcctttccaac
aagattatgaggatgaggtctgtatggttgatcttttgttacacatgatactacaactagagctcgacctttatatccaactatctg
taatcagtatcagttgtatgttattaattagtattattatatattgttttttttttcttcttacttttatagtaggaaatgttttattttctgatgt
actatttacaccaggtatactaccagctgatctgccttcacattcatagcgaaaacgtaaagctttgcttgctggttgttctattat
ttcaacataaggcaataatcgttctgtgttcatttctcttggaatttgatcttccctcccattatatttagtatcagtttcaatgacttc
aactatacaaattatgaaataaaatataatgtaaaaaataaaatgtaatttatgaaatttatgtaaaaatataataaaattttcaata
cttactgacatcactcatatgtatatttccatcactcatatcatggaattgttccatgcctcttactgtttaatacattgaataatagt
catctgaaaaatgatttcttatcatcttatgttattaaaataatttctattttattatatttctaatattatacagaattttttttaacaattcta
ttagaacaattatataaaatataaaatgattgtaaagtaacaatatatcaaaaaatttttttttattcttttatattttgaacactttcatc
aaagtgagagcaaaaattaactacatgatctaactcttgacagaaacttcataatattatgtagaaaaaatttcgcgcgcgttta
gttaagataaatggaaatataataattggacagtaataaatacctagaaagaaataaaatgtatagaaggaaaattgattttgg
gatcaatttttttgatgtaaagaaaattggatgttcataaattcatgaatcgttcatcttgatagaaaaatatatttatgtcgtaaaa
gatgtcgtatagatgatgattgatataaaacgtgttaggcgtaacttttgattaacactaacgagtgaatcaaacacatattaat
gaacatggataaaaatttgttaaatctctagcaaacgaaacgacagctgttcacaggtatacttctaaagacactgcacacaa
gtgtcccaagtcccaagggaagagaaacgagagtagagagtgaggagggatgccatgctgatccaatcattattttggga

aagcctagcttcgtacaagccgtgcagctataattaaactatcgtaggacaatgtgacgagaaagacttgcctgcagcttgat
caacatagtaaggcactttcgaatctgaaagcacgcgcactgttttggataattcttctactaggaaatgcttatattcctgaaat
ttttttataaaacacgcactgtgctaaataaattgaagaaacgcgatgtgttaatgtttagatctataattgaaattcttgtatttgtc
aaatattgctgcgcacactatttgcacgcgacataaaagcatgcacggccgtgactagagacaagactttatgcggcataac
attttgctcggactttgtatcggtactgacatgtccgttgcttttaatggtagaagcaaagcaatatgctatatacttcgcggata
cgcgcaagtacgcgtaccctgacggttctatactcacaaccaattcaaagtgagtgaacgtgtgttaacattatatactaccttt
cgattatttccttgaaatccatagatctaatgacgtcgaatttttaatatttaattcctaattcttaattctaattctcttcatatttttcat
attatcttttatattgattgcatttcttgttcttgcatatttttaagcttgagttgaaaaccactaataaattacgtatgttcagcgttctt
tggcataaatgtattaaatatcttatatctaaaataaatttgtgtctaaggttcagcgaatttatcatatgtagattaaaattttcagg
gacgtagataggttaccagaaactaggtacttgatgtcggatgagtcatgcagacacgtcacataattctgttttatcatgtga
aattgtagagcgttagataatcatatctatttacttgaaattatataaaatgttattgttttgttataaatattatgtccgataact

RNA9889
attaacaaactaaactaatatttttatggtcgtacctgaatatttttatatataatattcttttgatctggaattgattgggagcagaa
taaaaacacgtaatttttatcattaaataagtatgtatatatatatatagatcaatatattctgtatatatgtataagtcatacattatat
agatttttctttttcaaagcagtttataaaaatttgtattgccatacaaatattgcacataatttccgctcttattattgtattgctgaat
atttaaatacaatattttaatacaagagcaggatacttgtatattatttaaactgtaaaaattaagcccgcttttgtcaacgttattat
cattaacaacatacttttctcgtcgcattaatggatataaaaatgcatccattcgtgcaatcatcagtaataattacaaaaataca
attttctcttcaaatatacaaggccattgccatccaaaaatttaagtacaaattccaactatttatacaaaatacatttttaaaaaag
tataaaacaagattcttcagtgcgtgaaaatatatatatatatgtatatatatatatgaatatagtatttcttaatacaatgaaaattc
taattcatgaaaaaatatcaatctttgcacaacggaaatttttatcatttttttatgttaatcttctagattattaggcactattggac
gaaattcaacagacaaaatatataacaataagtataagaaatttttaaatttaaatatttccagtgtgcaaagatattagattaatt
atatagttagtatgacgtgacttaagagcgctgaaacatagatatttcgataaatatatattcgtgattcataaaaatgctgtaaa
cattctccaaatgtacaagttagtccaaatttttgggacttccaacaaatttttttagattcatcaatacaattattattattacaacaa
atagggttgataaaacccatcagaaaaatgcataacatgttgatgactcgcatcacaaagtcttgctaattttttttttcttgcatat
agaataaatgtgaacgcgattagtattacaaagaagtaaaatcagcaatttcgcataaatgtagcacaatgtacatttagaaat
tttgacgttgcgacgatgctaatgatatcatttaaacccactcccggcaaagctgtagaattgaagacaatttgataaaaaaata
ctcacgcatctcagcttctgcctgtacatgtctcaaaactagcgcgttttggctgttattaatcttttgtctctttcgccttaatgaat
caggatcatctataaatgattgtatattaaccacatgacttttaaatattaatatatcaatttttttctgaagacaacaaacctataaat
tttgatctaagtttttttaacctttaaaaatattatatctgaacaaatagttaagtcagtggttcaaaataagtttaagttacaaacaaa
taaaaaaatcttaaatataaattaattttaactataaattaagtgcttattctaagactagaaacaaagtgatgcctataaacttgga
acactatataaaaacttaacaatgtgtcataaatagtaaaaagcaatgcacaaaattaaagatatgtataataagtagcaacac
tataaaatttgactagaagaaacacaaactttgatgataaataacaagtttcagccgattttatatttcagctatttttttatataaaa
gactatagaaataaaataacaaatttgtactctgactagaaaaaatagtactgcattggtttcagagtatattaatcaaagtagc
ataataaaaattgctacatgtactttttttaaaaaatgaaaaaaatataaaatatatcttaagaacgcaaacacactcaagttaca
aaaatttcatattattatcgtaaatacgtaacttcattcaatcataatgctttttgtttctgaaaattagcaattatctgctataacatc
agcatttttctattataatatttgaaaaaaaatatactcttaaatggaagtaggctaaaaacacaataagacatattaaatctcctt
tttttcagaataaatctttccatcttccacaggactataatagaaagacatttcatcaaattcagaagcagttgcgtgcggcgcc
atagcagtatataatgcataatgttcagcttctgttaattccattccatatggtggtgtttcatcaccttccaatctaagtgattttac
gctagtagtacttactacactagctcttggaatttgtaattgtgaattatttgatgtattgtttgttgtcaaatatgaaatgtttcctgt
agtagtcaaagattgagtagtttctttggatacatttaaaattgtgtctttatctttttttcactttttttgcaaataattttgaaaataatc
cttgtttcgagtttttagttaaagtactaggtaaggaaggtaaagttttatttggtgcttcaatgaaagaatctggtgtagtatgac
aggatataggacgaggtaaaagaggcatagaaggcattttccggatacgtttcggtggtaaaggcggtaatctttcttccgtt
tcctgcgttccatctcttttcgtagtcactggcggggaaggacaagattgattagtatctgtttttagtacacacacatcttcatac

tttctttcgttcgataaatcggtaaattcaataggatttttcatagccatttgtaaactagtataagtctgattatcgcaaacatcaat
atccatagattgatcagttgtattctgttcaaatgctgcctgcactaactttgcatgtgtatcggcataaatttgatctaattctgct
acttgtgttaataaatcattgaaagacttattacaatcttctgtcacttcaattttaaaatctatttcattttcttctctttgagacatatt
tgtcctttttgtacccatttactcacttcagaataatcgaaccaatcagatttatcttttaatatttctgtatttttttaatacgcacgcgt
tttcaacagtattagatttaggacgagttaaaatttctaaattcatgtccttcgtttctttatctatataattgggataagatttgtaca
ttctatttaattgttcgttttctatagttcgtttctcttcattgattgataaatcattatttcggtaatctataatagtatttttagcatgatc
tatattttgtgcaatattttgatttatttccatacttccattataagaatctaatgtatttttttacattgtacaaatcattcaaagcacgta
acgcagaaattttattattccatttttttgtgtcgaagtcattattgttatattcgtcaatattacgcggaaatttcggagtaacgtttg
aaaacaatgtcggttcagtagctaaaattttgccaaatgaagtgtaatctgttttcttcctggcaaatgctttccgtaaagaccag
aaagcaggcctacctgcaccgagaggtaacatcagaaaaggaaatggttcgcttgttgcaccatctgatggtcttttaagttg
aatatatacttgcactggttgttccacttgctgcatacgataagtcggtgttctaaatgcaattgctgtctattaagataaatcaatt
tatcaaattaatattaataaagatacaaaatttaaataattttatataaaaatcgaatttcatacttgtttatgtacatgaacaggttg
aaaatcaccaaatccttcccaaagcacttgtccatctttttcttcaaagaatctaacttgtatatcttcttttgcaaccttctcacata
ataaaatcatttccataccaccagcgactgatgcgttactatgactgagtttgcatatcacaagatctgacattgcctcttaaata
tttaatatttaattatttaatacataatcgaataattgatatcttgataaaatataattaatctgaacatacttttatcaaatataggatc
ggatactactggttgtagtggcacattgaattttcttttttgtgatccttctaaaaagacttgaaagcataatctcactgcattgag
atcaatactagtaggttgtcttttgtgttcgaagccagctattaaaaaattaaatgtgatttgttttgtgccaaatatagttaaataa
atctactgaattttaatttacttcgaaaaggatctacacgaatttcttctcttattttaagcgcctcttcaatatctttttttttacacatt
gaattccaagatttgcaaatgtaactgtcatattctctgatgaaacttctactgtacaaacaccttgtttacatgcttcctttccaac
aagattatgaggatgaggtctgtatggttgatcttttgttacacatgatactacaactagagctcgacctttatatccaactatctg
taatcagtatcagttgtatgttattaattagtattattatatattgtttttttttcttcttacttttatagtaggaaatgttttattttctgatgt
actatttacaccaggtatactaccagctgatctgccttcacattcatagcgaaaacgtaaagctttgcttgctggttgttctattat
ttcaacataaggcaataatcgttctgtgttcatttctcttggaatttgatcttccctcccattatatttagtatcagtttcaatgacttc
aactatacaaattatgaaataaaatataatgtaaaaaataaaatgtaatttatgaaatttatgtaaaaatataataaaattttcaata
cttactgacatcactcatatgtatatttccatcactcatatcatggaattgttccatgcctcttactgtttaatacattgaataatagt
catctgaaaaatgatttcttatcatcttatgttattaaaataatttctattttattatatttctaatattatacagaattttttttaacaattcta
ttagaacaattatataaaatataaaatgattgtaaagtaacaatatatcaaaaaatttttttattcttttatatttgaacactttcatc
aaagtgagagcaaaaattaactacatgatctaactcttgacagaaacttcataatattatgtagaaaaaatttcgcgcgcgttta
gttaagataaatggaaatataataattggacagtaataaatacctagaaagaaataaaatgtatagaaggaaaattgattttgg
gatcaattttttgatgtaaagaaaattggatgttcataaattcatgaatcgttcatcttgatagaaaaatatatttatgtcgtaaaa
gatgtcgtatagatgatgattgatataaaacgtgttaggcgtaacttttgattaacactaacgagtgaatcaaacacatattaat
gaacatggataaaaatttgttaaatctctagcaaacgaaacgacagctgttcacaggtatacttctaaagacactgcacacaa
gtgtcccaagtcccaagggaagagaaacgagagtagagagtgaggagggatgccatgctgatccaatcattattttggga
aagcctagcttcgtacaagccgtgcagctataattaaactatcgtaggacaatgtgacgagaaagacttgcctgcagcttgat
caacatagtaaggcactttcgaatctgaaagcacgcgcactgttttggataattcttctactaggaaatgcttatattcctgaaat
tttttataaaacacgcactgtgctaaataaattgaagaaacgcgatgtgttaatgtttagatctataattgaaattcttgtatttgtc
aaatattgctgcgcacactatttgcacgcgacataaaagcatgcacggccgtgactagagacaagactttatgcggcataac
attttgctcggactttgtatcggtactgacatgtccgttgcttttaatggtagaagcaaagcaatatgctatatacttcgcggata
cgcgcaagtacgcgtaccctgacggttctatactcacaaccaattcaaagtgagtgaacgtgtgttaacattatatactaccttt
cgatt

RNA9890

attaacaaactaaactaatatttttatggtcgtacctgaatatttttttatatataatattcttttgatctggaattgattgggagcagaa
taaaaacacgtaatttttatcattaaataagtatgtatatatatatatagatcaatatattctgtatatatgtataagtcatacattatat

agatttttctttttcaaagcagtttataaaaatttgtattgccatacaaatattgcacataatttccgctcttattattgtattgctgaat
atttaaatacaatattttaatacaagagcaggatacttgtatattatttaaactgtaaaaattaagcccgcttttgtcaacgttattat
cattaacaacatacttttctcgtcgcattaatggatataaaaatgcatccattcgtgcaatcatcagtaataattacaaaaataca
attttctcttcaaatatacaaggccattgccatccaaaaatttaagtacaaattccaactatttatacaaaatacatttttaaaaaag
tataaaacaagattcttcagtgcgtgaaaatatatatatatatgtatatatatatatgaatatagtatttcttaatacaatgaaaattc
taattcatgaaaaaatatcaatctttgcacaacggaaatttttttatcatttttttatgttaatcttctagattattaggcactattggac
gaaattcaacagacaaaatatataacaataagtataagaaatttttaaatttaaatatttccagtgtgcaaagatattagattaatt
atatagttagtatgacgtgacttaagagcgctgaaacatagatatttcgataaatatatattcgtgattcataaaaatgctgtaaa
cattctccaaatgtacaagttagtccaaatttttgggacttccaacaaatttttttagattcatcaatacaattattattattacaacaa
atagggttgataaaacccatcagaaaaatgcataacatgttgatgactcgcatcacaaagtcttgctaatttttttttcttgcatat
agaataaatgtgaacgcgattagtattacaaagaagtaaaatcagcaatttcgcataaatgtagcacaatgtacatttagaaat
tttgacgttgcgacgatgctaatgatatcatttaaacccactcccggcaaagctgtagaattgaagacaatttgataaaaaata
ctcacgcatctcagcttctgcctgtacatgtctcaaaactagcgcgttttggctgttattaatcttttgtctctttcgccttaatgaat
caggatcatctataaatgattgtatattaaccacatgacttttaaatattaatatatcaatttttttctgaagacaacaaacctataaat
tttgatctaagttttttaacctttaaaaatattatatctgaacaaatagttaagtcagtggttcaaaataagtttaagttacaaacaaa
taaaaaatcttaaatataaattaattttaactataaattaagtgcttattctaagactagaaacaaagtgatgcctataaacttgga
acactatataaaaacttaacaatgtgtcataaatagtaaaaagcaatgcacaaaattaaagatatgtataataagtagcaacac
tataaaatttgactagaagaaacacaaactttgatgataaataacaagtttcagccgattttatatttcagctatttttttatataaaa
gactatagaaataaaataacaaatttgtactctgactagaaaaaatagtactgcattggtttcagagtatattaatcaaagtagc
ataataaaaattgctacatgtactttttttaaaaaatgaaaaaaatataaaatatatcttaagaacgcaaacacactcaagttaca
aaaatttcatattattatcgtaaatacgtaacttcattcaatcataatgctttttgtttctgaaaattagcaattatctgctataacatc
agcatttttctattataatatttgaaaaaaaatatactcttaaatggaagtaggctaaaaacacaataagacatattaaatctcctt
tttttcagaataaatctttccatcttccacaggactataatagaaagacatttcatcaaattcagaagcagttgcgtgcggcgcc
atagcagtatataatgcataatgttcagcttctgttaattccattccatatggtggtgtttcatcaccttccaatctaagtgattttac
gctagtagtacttactacactagctcttggaatttgtaattgtgaattatttgatgtattgtttgttgtcaaatatgaaatgtttcctgt
agtagtcaaagattgagtagtttctttggatacatttaaaattgtgtctttatcttttttcactttttttgcaaataattttgaaaataatc
cttgtttcgagtttttagttaaagtactaggtaaggaaggtaaagttttatttggtgcttcaatgaaagaatctggtgtagtatgac
aggatataggacgaggtaaaagaggcatagaaggcattttccggatacgtttcggtggtaaaggcggtaatctttcttccgtt
tcctgcgttccatctcttttcgtagtcactggcggggaaggacaagattgattagtatctgtttttagtacacacacatcttcatac
tttctttcgttcgataaatcggtaaattcaataggatttttcatagccatttgtaaactagtataagtctgattatcgcaaacatcaat
atccatagattgatcagttgtattctgttcaaatgctgcctgcactaactttgcatgtgtatcggcataaatttgatctaattctgct
acttgtgttaataaatcattgaaagacttattacaatcttctgtcacttcaattttaaaatctatttcattttctttctctttgagacatatt
tgtcctttttgtacccatttactcacttcagaataatcgaaccaatcagatttatcttttaatatttctgtatttttttaatacgcacgcgt
tttcaacagtattagatttaggacgagttaaaatttctaaattcatgtccttcgtttctttatctatataattgggataagatttgtaca
ttctatttaattgttcgttttctatagttcgtttctcttcattgattgataaatcattatttcggtaatctataatagtatttttagcatgatc
tatattttgtgcaatattttgatttatttccatacttccattataagaatctaatgtattttttacattgtacaaatcattcaaagcacgta
acgcagaaattttattattccattttttgtgtcgaagtcattattgttatattcgtcaatattacgcggaaatttcggagtaacgtttg
aaaacaatgtcggttcagtagctaaaattttgccaaatgaagtgtaatctgttttcttcctggcaaatgctttccgtaaagaccag
aaagcaggcctacctgcaccgagaggtaacatcagaaaaggaaatggttcgcttgttgcaccatctgatggtcttttaagttg
aatatatacttgcactggttgttccacttgctgcatacgataagtcggtgttctaaatgcaattgctgtctattaagataaatcaatt
tatcaaattaatattaataaagatacaaaatttaaataattttatataaaaatcgaatttcatacttgtttatgtacatgaacaggttg
aaaatcaccaaatccttcccaaagcacttgtccatctttttcttcaaagaatctaacttgtatatcttcttttgcaaccttctcacata
ataaaatcatttccataccaccagcgactgatgcgttactatgactgagtttgcatatcacaagatctgacattgcctcttaaata
tttaatatttaattatttaatacataatcgaataattgatatcttgataaaatataattaatctgaacatacttttatcaaatataggatc

ggatactactggttgtagtggcacattgaattttctttttgtgatccttctaaaaagacttgaaagcataatctcactgcattgag
atcaatactagtaggttgtcttttgtgttcgaagccagctattaaaaaattaaatgtgatttgttttgtgccaaatatagttaaataa
atctactgaattttaatttacttcgaaaaggatctacacgaatttcttctcttattttaagcgcctcttcaatatctttttttttacacatt
gaattccaagatttgcaaatgtaactgtcatattctctgatgaaacttctactgtacaaacaccttgtttacatgcttcctttccaac
aagattatgaggatgaggtctgtatggttgatcttttgttacacatgatactacaactagagctcgacctttatatccaactatctg
taatcagtatcagttgtatgttattaattagtattattatatattgtttttttttcttcttacttttatagtaggaaatgttttattttctgatgt
actatttacaccaggtatactaccagctgatctgccttcacattcatagcgaaaacgtaaagctttgcttgctggttgttctattat
ttcaacataaggcaataatcgttctgtgttcatttctcttggaatttgatcttccctcccattatatttagtatcagtttcaatgacttc
aactatacaaattatgaaataaaatataatgtaaaaaataaaatgtaatttatgaaatttatgtaaaaatataataaaattttcaata
cttactgacatcactcatatgtatatttccatcactcatatcatggaattgttccatgcctcttactgtttaatacattgaataatagt
catctgaaaaatgatttcttatcatcttatgttattaaaataatttctattttattatatttctaatattatacagaattttttttaacaattcta
ttagaacaattatataaaatataaaatgattgtaaagtaacaatatatcaaaaaatttttttttattcttttatattttgaacactttcatc
aaagtgagagcaaaaattaactacatgatctaactcttgacagaaacttcataatattatgtagaaaaaaatttcgcgcgcgttta
gttaagataaatggaaatataataattggacagtaataaatacctagaaagaaataaaatgtatagaaggaaaattgattttgg
gatcaattttttttgatgtaaagaaaattggatgttcataaattcatgaatcgttcatcttgatagaaaaatatatttatgtcgtaaaa
gatgtcgtatagatgatgattgatataaaacgtgttaggcgtaacttttgattaacactaacgagtgaatcaaacacatattaat
gaacatggataaaaatttgttaaatctctagcaaacgaaacgacagctgttcacaggtatacttctaaagacactgcacacaa
gtgtcccaagtcccaagggaagagaaacgagagtagagagtgaggagggatgccatgctgatccaatcattattttggga
aagcctagcttcgtacaagccgtgcagctataattaaactatcgtaggacaatgtgacgagaaag

RNA9891
attaacaaactaaactaatatttttatggtcgtacctgaatatttttttatatataatattcttttgatctggaattgattgggagcagaa
taaaaacacgtaatttttatcattaaataagtatgtatatatatatatagatcaatatattctgtatatatgtataagtcatacattatat
agatttttctttttcaaagcagtttataaaaatttgtattgccatacaaatattgcacataatttccgctcttattattgtattgctgaat
atttaaatacaatattttaatacaagagcaggatacttgtatattatttaaactgtaaaaattaagcccgcttttgtcaacgttattat
cattaacaacatacttttctcgtcgcattaatggatataaaaatgcatccattcgtgcaatcatcagtaataattacaaaaataca
attttctcttcaaatatacaaggccattgccatccaaaaatttaagtacaaattccaactatttatacaaaatacatttttaaaaaag
tataaaacaagattcttcagtgcgtgaaaatatatatatatatgtatatatatatatgaatatagtatttcttaatacaatgaaaattc
taattcatgaaaaaatatcaatctttgcacaacggaaattttttatcatttttttatgttaatcttctagattattaggcactattggac
gaaattcaacagacaaaatatataacaataagtataagaaatttttaaatttaaatatttccagtgtgcaaagatattagattaatt
atatagttagtatgacgtgacttaagagcgctgaaacatagatatttcgataaatatatattcgtgattcataaaaatgctgtaaa
cattctccaaatgtacaagttagtccaaatttttgggacttccaacaaatttttttagattcatcaatacaattattattattacaacaa
atagggttgataaaacccatcagaaaaatgcataacatgttgatgactcgcatcacaaagtcttgctaattttttttttcttgcatat
agaataaatgtgaacgcgattagtattacaaagaagtaaaatcagcaatttcgcataaatgtagcacaatgtacatttagaaat
tttgacgttgcgacgatgctaatgatatcatttaaacccactcccggcaaagctgtagaattgaagacaatttgataaaaaata
ctcacgcatctcagcttctgcctgtacatgtctcaaaactagcgcgttttggctgttattaatcttttgtctctttcgccttaatgaat
caggatcatctataaatgattgtatattaaccacatgacttttaaatattaatatatcaatttttttctgaagacaacaaacctataaat
tttgatctaagttttttaacctttaaaaatattatatctgaacaaatagttaagtcagtggttcaaaataagtttaagttacaaacaaa
taaaaaatcttaaatataaattaattttaactataaattaagtgcttattctaagactagaaacaaagtgatgcctataaacttgga
acactatataaaaacttaacaatgtgtcataaatagtaaaaagcaatgcacaaaattaaagatatgtataataagtagcaacac
tataaaatttgactagaagaaacacaaactttgatgataaataacaagtttcagccgattttatatttcagctattttttttatataaaa
gactatagaaataaaataacaaatttgtactctgactagaaaaaatagtactgcattggtttcagagtatattaatcaaagtagc
ataataaaaattgctacatgtacttttttttaaaaaatgaaaaaaatataaaatatatcttaagaacgcaaacacactcaagttaca
aaaatttcatattattatcgtaaatacgtaacttcattcaatcataatgctttttgtttctgaaaattagcaattatctgctataacatc

agcatttttctattataatatttgaaaaaaaatatactcttaaatggaagtaggctaaaaacacaataagacatattaaatctcctt
tttttcagaataaatctttccatcttccacaggactataatagaaagacatttcatcaaattcagaagcagttgcgtgcggcgcc
atagcagtatataatgcataatgttcagcttctgttaattccattccatatggtggtgtttcatcaccttccaatctaagtgattttac
gctagtagtacttactacactagctcttggaatttgtaattgtgaattatttgatgtattgtttgttgtcaaatatgaaatgtttcctgt
agtagtcaaagattgagtagtttctttggatacatttaaaattgtgtctttatctttttcactttttttgcaaataattttgaaaataatc
cttgtttcgagtttttagttaaagtactaggtaaggaaggtaaagttttatttggtgcttcaatgaaagaatctggtgtagtatgac
aggatataggacgaggtaaaagaggcatagaaggcattttccggatacgtttcggtggtaaaggcggtaatctttcttccgtt
tcctgcgttccatctcttttcgtagtcactggcggggaaggacaagattgattagtatctgtttttagtacacacacatcttcatac
tttctttcgttcgataaatcggtaaattcaataggatttttcatagccatttgtaaactagtataagtctgattatcgcaaacatcaat
atccatagattgatcagttgtattctgttcaaatgctgcctgcactaactttgcatgtgtatcggcataaatttgatctaattctgct
acttgtgttaataaatcattgaaagacttattacaatcttctgtcacttcaattttaaaatctatttcattttctttctctttgagacatatt
tgtccttttttgtacccatttactcacttcagaataatcgaaccaatcagatttatcttttaatatttctgtattttttaatacgcacgcgt
tttcaacagtattagatttaggacgagttaaaatttctaaattcatgtccttcgtttctttatctatataattgggataagatttgtaca
ttctatttaattgttcgttttctatagttcgtttctcttcattgattgataaatcattatttcggtaatctataatagtatttttagcatgatc
tatattttgtgcaatattttgatttatttccatacttccattataagaatctaatgtattttttacattgtacaaatcattcaaagcacgta
acgcagaaattttattattccattttttgtgtcgaagtcattattgttatattcgtcaatattacgcggaaatttcggagtaacgtttg
aaaacaatgtcggttcagtagctaaaattttgccaaatgaagtgtaatctgttttcttcctggcaaatgctttccgtaaagaccag
aaagcaggcctacctgcaccgagaggtaacatcagaaaaggaaatggttcgcttgttgcaccatctgatggtcttttaagttg
aatatatacttgcactggttgttccacttgctgcatacgataagtcggtgttctaaatgcaattgctgtctattaagataaatcaatt
tatcaaattaatattaataaagatacaaaatttaaataattttatataaaaatcgaatttcatacttgtttatgtacatgaacaggttg
aaaatcaccaaatccttcccaaagcacttgtccatctttttcttcaaagaatctaacttgtatatcttcttttgcaaccttctcacata
ataaaatcatttccataccaccagcgactgatgcgttactatgactgagtttgcatatcacaagatctgacattgcctcttaaata
tttaatatttaattatttaatacataatcgaataattgatatcttgataaaatataattaatctgaacatacttttatcaaatataggatc
ggatactactggttgtagtggcacattgaattttcttttttgtgatccttctaaaaagacttgaaagcataatctcactgcattgag
atcaatactagtaggttgtcttttgtgttcgaagccagctattaaaaaattaaatgtgatttgttttgtgccaaatatagttaaataa
atctactgaattttaatttacttcgaaaaggatctacacgaatttcttctcttattttaagcgcctcttcaatatcttttttttttacacatt
gaattccaagatttgcaaatgtaactgtcatattctctgatgaaacttctactgtacaaacaccttgtttacatgcttcctttccaac
aagattatgaggatgaggtctgtatggttgatcttttgttacacatgatactacaactagagctcgacctttatatccaactatctg
taatcagtatcagttgtatgttattaattagtattattatatattgtttttttttcttcttacttttatagtaggaaatgttttattttctgatgt
actatttacaccaggtatactaccagctgatctgccttcacattcatagcgaaaacgtaaagctttgcttgctggttgttctattat
ttcaacataaggcaataatcgttctgtgttcatttctcttggaat
